# Discovery of VU6025733 (AG06827): A Highly Selective, Orally Bioavailable, and Structurally Distinct M_4_ Muscarinic Acetylcholine Receptor Positive Allosteric Modulator (PAM) with Robust *In Vivo* Efficacy

**DOI:** 10.1021/acschemneuro.5c00963

**Published:** 2026-01-25

**Authors:** Alison R. Gregro, Charlotte Park, Madeline F. Long, Logan A. Baker, Katrina A. Bollinger, Anna E. Ringuette, Li Peng, Vincent B. Luscombe, Natasha B. Billard, Alice L. Rodriguez, Colleen M. Niswender, Weimin Peng, Jonathan W. Dickerson, Jerri M. Rook, Jordan O’Neill, Sichen Chang, Harrie C. M. Boonen, Thomas Jensen, Morten S. Thomsen, Thomas M. Bridges, Olivier Boutaud, P. Jeffrey Conn, Darren W. Engers, Craig W. Lindsley, Kayla J. Temple

**Affiliations:** † Warren Center for Neuroscience Drug Discovery, 5718 Vanderbilt University, Nashville, Tennessee 37232, United States; ‡ Department of Pharmacology, 12328 Vanderbilt University School of Medicine, Nashville, Tennessee 37232, United States; § Department of Chemistry, Vanderbilt University, Nashville, Tennessee 37232, United States; ∥ Department of Biochemistry, Vanderbilt University, Nashville, Tennessee 37232, United States; ⊥ Vanderbilt Kennedy Center, Vanderbilt University School of Medicine, Nashville, Tennessee 37232, United States; # Vanderbilt Brain Institute, Vanderbilt University School of Medicine, Nashville, Tennessee 37232, United States; ∇ Neuroscience Drug Discovery Denmark, 8134 H. Lundbeck A/S, 9 Ottiliavej, Valby, Copenhagen DK-2500, Denmark; ○ Vanderbilt Institute for Therapeutic Advances, Vanderbilt University, Nashville, Tennessee 37232, United States

**Keywords:** muscarinic acetylcholine receptor (mAChR), M_4_, Positive allosteric modulator (PAM), structure−activity relationship (SAR), schizophrenia, parkinson’s disease, Alzheimer’s disease

## Abstract

This work describes progress toward an M_4_ PAM preclinical candidate. The SAR to address potency, clearance, subtype selectivity, CNS exposure, and P-gp efflux are detailed within. A novel 1-(7,8-dimethyl-[1,2,4]­triazolo­[4,3-*b*]­pyridazin-6-yl)­piperidin-4-ol scaffold was identified, and optimization provided a highly potent analog **VU6025733** (hM_4_ EC_50_ = 23 nM; rM_4_ EC_50_ = 55 nM). Further characterization revealed a highly selective compound across muscarinic acetylcholine receptor subtypes with exceptional DMPK properties (*in vivo* rat CL_p_ = 5.9 mL/min/kg; *t*_1/2_ = 4.8 h; CYP1A2 & CYP2C9 IC_50_s > 30 μM, CYP2D6 IC_50_ > 9 μM; CYP3A4 IC_50_ > 25 μM). Moreover, **VU6025733** demonstrated robust *in vivo* efficacy in a rat amphetamine-induced hyperlocomotion model in a dose-dependent manner. However, hepatotoxicity risk precluded further development.

## Graphical abstract

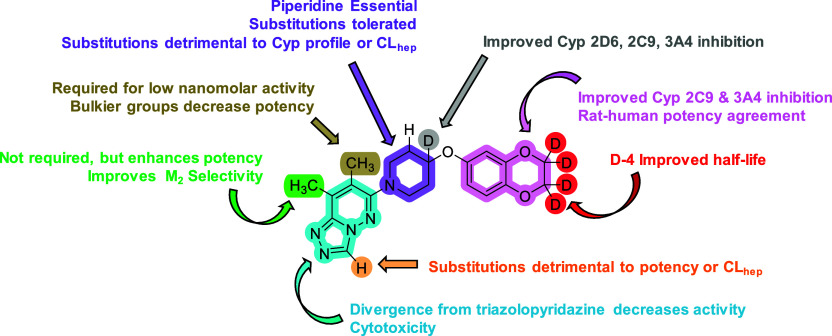

## Introduction

Cholinergic neurotransmission involves the binding of an orthosteric endogenous agonist, acetylcholine (ACh), to activate receptors such as the nicotinic acetylcholine (nAChRs) or muscarinic acetylcholine receptors (mAChRs).^1^ Often conditions involving cognitive impairment, e.g., Alzheimer’s disease (AD), have been associated with lower levels of acetylcholine in the brain. Such reductions in the levels of acetylcholine are perceived to be a consequence of the deterioration of cholinergic neurons of the basal forebrain, which widely innervates multiple areas of the brain crucial for higher processes.^2,3^ Moreover, cholinergic hypofunction has been clinically linked to cognitive deficits in patients suffering from schizophrenia.^4^

Previous endeavors to increase acetylcholine levels have taken one of two approaches: (1) increasing the levels of the acetylcholine precursor, choline; or (2) inhibition of the enzyme responsible for the metabolism of acetylcholine, acetylcholinesterase (AChE). Attempts to augment central cholinergic function through administration of choline or phosphatidylcholine have proved futile.^5^ Nonetheless, AChE inhibitors (AChEI’s) have been approved for the use in palliative, but not disease-modifying, treatments of cognitive deficits in Alzheimer’s patients.^6^ While AChEI’s have demonstrated therapeutic efficacy, they induce cholinergic side effects due to peripheral acetylcholine stimulation. These side effects have been observed in nearly one-third of the patients treated and include abdominal cramps, nausea, vomiting, and diarrhea.^7^ Additionally, some AChEI’s, such as tacrine, cause significant hepatotoxicity with elevated liver transaminases in nearly 30% of patients treated.^8^ Such adverse effects greatly hinder the clinical utility of AChEI’s and highlight the glaring need for an alternative approach to pharmacologically target cholinergic hypofunction. One such approach, as showcased in this paper, is to target the activation of mAChRs, which are widely expressed throughout the body, including the brain.^9^

Muscarinic acetylcholine receptors are members of the Class A family of G-protein coupled receptors (GPCRs) and include five subtypes of receptors, designated M_1_ - M_5_.^10^ The subtypes can be grouped into two main categories: (1) those that are mainly G_q_-coupled and activate Phospholipase C (M_1_, M_3_, and M_5_) and (2) those that mainly couple to G_i/o_ and effector systems (M_2_ and M_4_).^11–13^ Not only are these five distinct mAChR subtypes prevalent and differentially expressed in the mammalian central nervous system, but they also play varying roles in cognitive, sensory, motor, and autonomic functions. Thus, it has been hypothesized that selective agonists of mAChR subtypes involved in regulating processes associated with cognitive function could provide a superior avenue in the treatment of psychosis, schizophrenia, and related disorders.

The activation of peripheral M_2_ and M_3_ mAChRs has been linked to the most prominent side effects of AChE inhibitors and other cholinergic agents (i.e., bradycardia, GI stress, and excessive salivation and sweating).^14,15^ Alternatively, the muscarinic M_4_ receptor has been demonstrated as playing a major role in cognitive processing and is viewed as the most plausible subtype for mediating the effects of mAChR dysfunction in psychotic disorders, including cognition disorders, neuropathic pain, and schizophrenia.^16–19^ As a result, considerable effort has been put forth to develop selective M_4_ agonists for the treatment of such disorders; however, attempts have fallen short due to the inability to design and develop highly selective compounds for mAChR M_4_. Past shortcomings can be attributed to targeting the highly conserved orthostatic ACh binding site.

Further target validation by Eli Lilly and Co., in collaboration with Novo Nordisk, came with the development of xanomeline (an M_1_/M_4_ preferring agonist) which further solidified the mACh system as a mechanism for treating psychosis and behavioral disturbances observed in both schizophrenia and AD patients.^20,21^ However, due to peripherally mediated cholinergic side effects which were attributed to the lack of mAChR selectivity, xanomeline’s clinical development was discontinued. To overcome these adverse events, Karuna Therapeutics (acquired by Bristol Myers Squibb) developed KarXT (Cobenfy) which was recently approved by the FDA as the first antipsychotic drug for the treatment of schizophrenia which targets the cholinergic receptors.^22^ Cobenfy is a treatment that coadministers xanomeline with trospium chloride (a peripherally restricted, pan-selective mAChR antagonist) which aids in minimizing the cholinergic adverse events observed when xanomeline is administered alone.^23^

To circumvent issues arising from targeting the highly conserved orthostatic binding site (e.g., lack of subtype selectivity), our approach is to develop compounds that act at allosteric sites of mAChRs that are less likely to be highly conserved. Allosteric activators can include (1) allosteric agonists, which directly activate the receptor in the absence of ACh at a site removed from the orthosteric site and (2) positive allosteric modulators (PAMs), which do not activate the receptor directly but potentiate activation of the receptor by the endogenous orthosteric agonist, ACh.^24,25^ It should be noted that it is possible for a single molecule to have both allosteric potentiator and allosteric agonist activity. It has been reported that a selective M_4_ PAM not only demonstrated robust efficacy in preclinical models of antipsychotic-like activity and enhancement of cognition but also, and perhaps more importantly, lacked the adverse cholinergic-related side effects previously observed with xanomeline.^26^ Therefore, one strategy to improve tolerability and safety profiles is the development of receptor-subtype-selective M_4_ PAMs. Cerevel Therapeutics (acquired by AbbVie) developed selective M_4_ PAM CVL-231 (Emraclidine), which is undergoing clinical investigation as an adjunct treatment for schizophrenia and neurodegenerative psychosis.^27^

Despite advances in mAChR research, there is still a scarcity of potent, efficacious, and selective activators of M_4_ mAChR that are also effective in the treatment of neurological and psychiatric disorders associated with cholinergic activity and diseases in which the muscarinic M_4_ receptor is involved. This paper details a recent effort to develop one such compound, **VU6025733**, a muscarinic M_4_ PAM.

## Results and Discussion

### Synthesis and SAR

We began our current effort with a high-throughput screen (HTS) identifying **VU0641491** and **VU0641483** as weak M_4_ PAMs with potencies in the micromolar range (Figure 1). Following an investigation into recent research in the field, we came across a structurally similar series which incorporates a 2-methoxy-5-(piperidin-4-yloxy)­pyridine moiety.^28^ Our first step was to replace the (4-fluorophenyl)­(piperazin-1-yl)­methanone tail of **VU0641491** and **VU0641483** with the 2-methoxy-5-(piperidin-4-yloxy)­pyridine moiety (Figure 1, shown in red) while retaining our [1,2,4]­triazolo­[4,3-*b*]­pyridazine headgroup (Figure 1, shown in blue). This resulted in lead compounds **VU6015863** and **VU6020378**, which displayed much improved human M_4_ (hM_4_) potency profiles when compared to predecessors **VU0641491** and **VU0641483** (18–52-fold). Unfortunately, this first-generation iteration suffered from many shortcomings, such as human-rat potency discrepancies typically 4–5 times less potent when screened against rat M_4_ (rM_4_), moderate to high predicted human hepatic clearance (CL_hep_), and/or inhibition of cytochrome P_450_s (CYPs).

**Figure 1:**
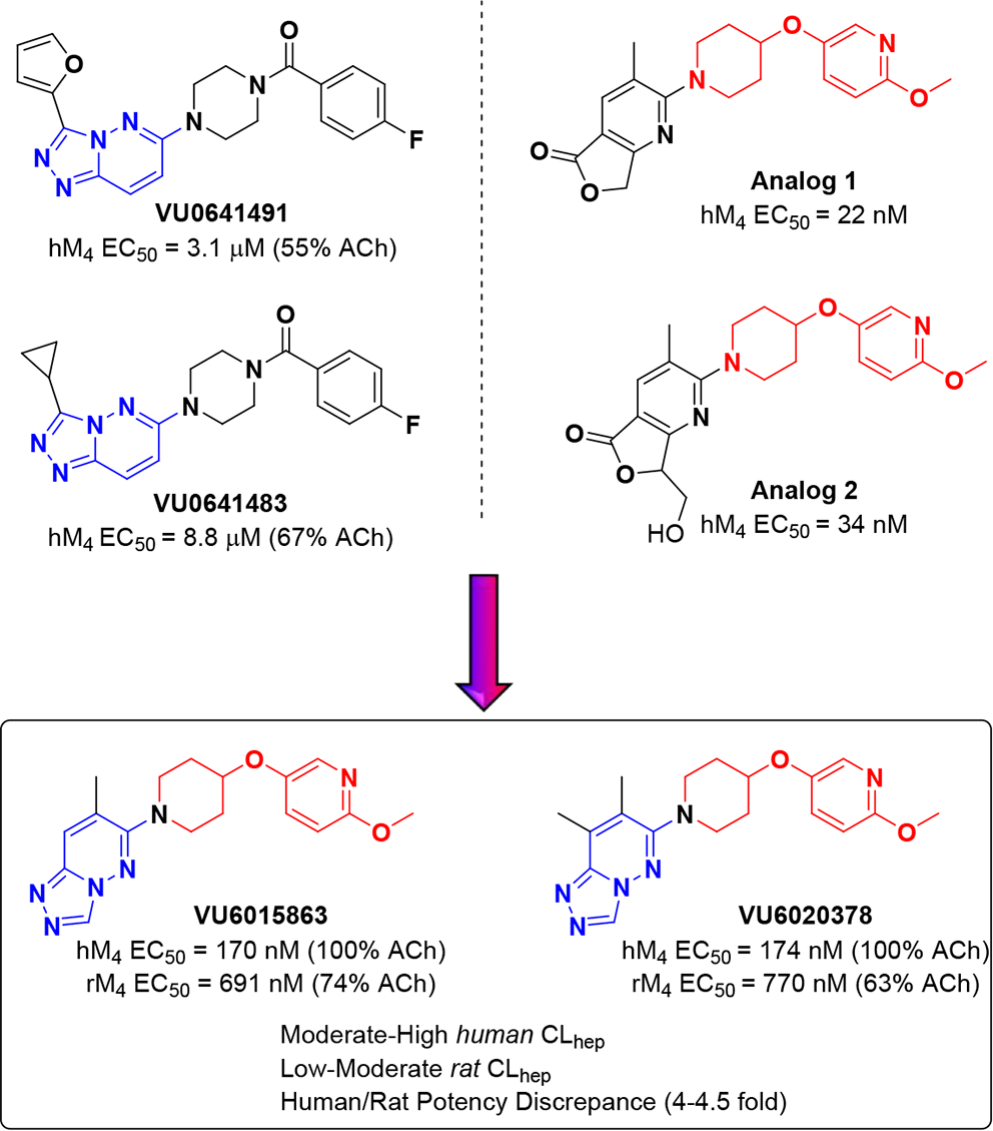
Scaffold hybridization provided a novel chemotype for the first-generation M_4_ PAM analogs.

To overcome these hurdles, we devised a multidimensional optimization approach summarized in Figure 2. We began our investigation with the substitution of the 2-methoxypyridine group of the ether linkage to generate our second generation of analogs (Table 1). These analogs were synthesized according to Scheme 1. In general, commercial alcohol **1** was converted into mesylate **2** and followed by nucleophilic substitution with various commercial alcohols and subsequent Boc-deprotection yielded intermediates **3**. The 6-chloro-[1,2,4]­triazolo­[4,3-*b*]­pyridazines **4** could then undergo nucleophilic aromatic substitution (S_N_Ar) with free amines **3** to give final compounds **5** or **6**. Select analogs were screened against hM_4_ to determine PAM activity with results highlighted in Table 1. The 4-fluoro benzene derivatives (**5a**: hM_4_ EC_50_ = 152 nM; **6a**: hM_4_ EC_50_ = 131 nM), while highly potent in relation to hM_4_, still suffered from a ∼3–4-fold human-rat M_4_ discrepancy (**5a**: rM_4_ EC_50_ = 605 nM; **6a**: rM_4_ EC_50_ = 324 nM). It also became apparent that the location of the fluoro-substituent was greatly important, as analogs with a 3-fluorophenyl were well tolerated (**5b**, **6b**, **5d**, and **6d**; hM_4_ EC_50_ ∼240–330 nM) as opposed to 2-fluorophenyl analogs which displayed a great reduction in potency (**5c**, **6c**, **5e**, and **6e**; hM_4_ EC_50_ > 1.4 μM). Both the *m*-methylphenyl (**5h**: hM_4_ EC_50_ = 160 nM, rM_4_ EC_50_ = 575 nM; and **6h**: hM_4_ EC_50_ = 159 nM, rM_4_ EC_50_ = 482 nM) and *p*-methylphenyl (**5g**: hM_4_ EC_50_ = 324 nM, rM_4_ EC_50_ = 893 nM; and **6g**: hM_4_ EC_50_ = 280 nM, rM_4_ EC_50_ = 985 nM) were tolerated; however, the *m*-methylphenyl derivatives were ∼2-fold more potent in both rM_4_ and hM_4_. Interestingly, introduction of a nitrogen *meta* to the ether linkage to generate pyridines **5i**, **6i**, **5j**, and **6j** (similar to lead compounds **VU6015863** and **VU6020378**) led to diminished activity; however, this phenomena was less pronounced when a 7,8-dimethyl-[1,2,4]­triazolo­[4,3-*b*]­pyridazine headgroup (R^1^ = Me) was employed (**6g** vs **6j**; ∼1.3-fold loss of activity) versus when a 7-methyl-[1,2,4]­triazolo­[4,3-*b*]­pyridazine headgroup (R^1^ = H) was present (**5g** vs **5j**; ∼2.7-fold loss of activity). Even more detrimental was the pyridine analog in which the nitrogen was *ortho* to the ether linkage (**5k**), resulting in significant loss of activity. Additional SAR revealed that exchanging the *p*-methylphenyl (**5g** and **6g**) moiety with 4-trifluormethylphenyl resulted in a loss of potency (**5f** and **6f**), although, once again, this phenomenon was less severe when a 7,8-dimethyl -[1,2,4]­triazolo­[4,3-*b*]­pyridazine headgroup was utilized (**6f**). Other R^3^ groups that provided low nanomolar compounds included the benzo­[*d*]­thiazole analogs (**5l**: hM_4_ EC_50_ = 80 nM; and **6l**: hM_4_ EC_50_ = 68 nM) and the 1-methyl-1*H*-indazole analogs (**5m**: hM_4_ EC_50_ = 216 nM; and **6m**: hM_4_ EC_50_ = 180 nM); however, these compounds were not pursued as they were shown to inhibit a multitude of CYP enzymes including CYP2C9, CYP2D6, and CYP3A4 (Table 2). Moreover, the benzo­[*d*]­thiazole analogs also displayed moderate to high predicted human hepatic clearance. We also examined naphthalene as a substitute to the 2-methoxypyridine group which resulted in a loss of hM_4_ PAM potency (**5o** and **6o**: hM_4_ EC_50_s > 1.1 μM). Intriguingly, introducing flexibility into the bicycle to give the 1,2,3,4-tetrahydronaphthalene analogs **5p** and **6p** enhanced potency by 2–2.5-fold. Further exploration led to the discovery of the 2,3-dihydrobenzo­[*b*]­[1,4]­dioxine analogs **5q** (hM_4_ EC_50_ = 134 nM, rM_4_ EC_50_ = 48 nM) and **6q (**hM_4_ EC_50_ = 38 nM, rM_4_ EC_50_ = 95 nM), both of which displayed low nanomolar potencies in hM_4_ and rM_4_. The addition of an extra methyl group on the triazolopyridazine headgroup (R^1^ = Me) of **6q** provided us with an analog with an EC_50_ < 100 nM in both human and rat M_4_ as well as improved rat and human predicted CL_hep_ (Table 2). Unfortunately, **6q** suffered from a short *in vivo* elimination half-life (*t*_1/2=_ 0.6 h), suggesting potential extrahepatic clearance mechanisms.

**Figure 2:**
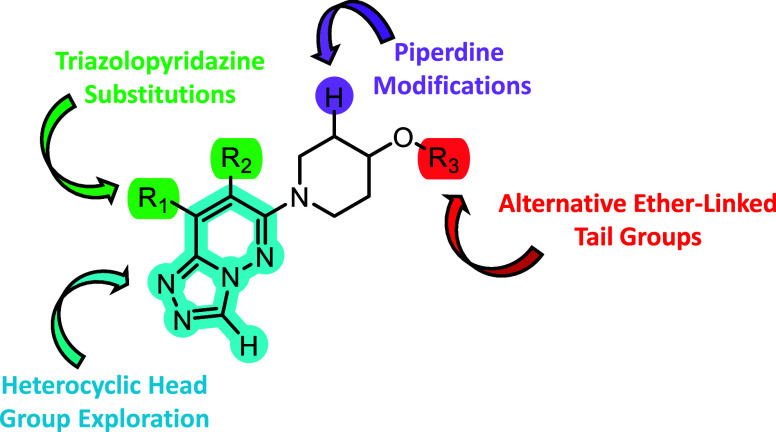
Library optimization strategy to improve M_4_ PAM potency as well as DMPK properties.

**Table 1:**
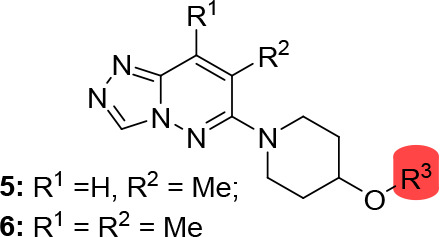

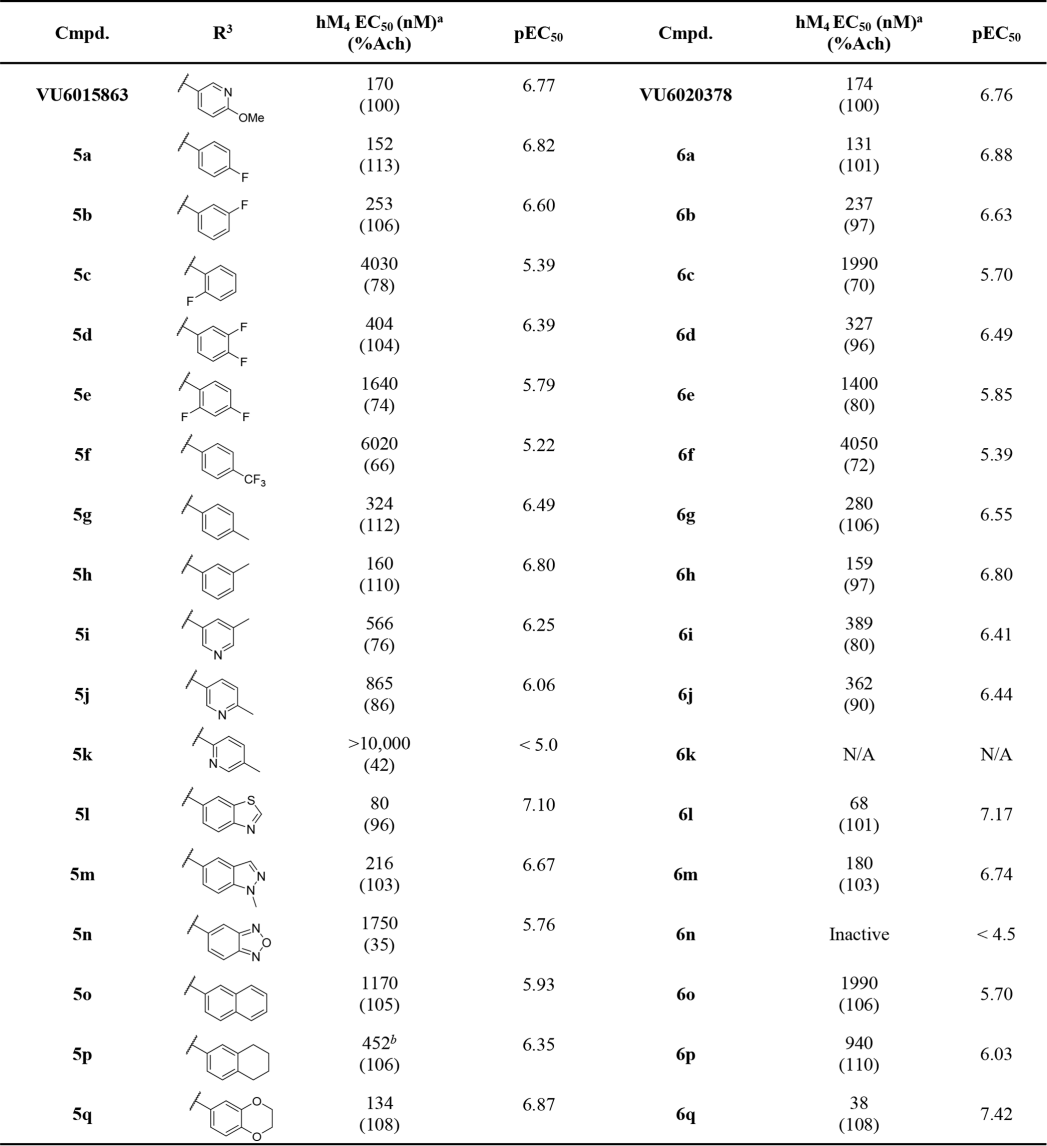
SAR of Second Generation M_4_ PAM Analogs **5** or 6^a^

a Calcium mobilization assays with hM_4/Gqi5_-CHO cells performed in the presence of an EC_20_ fixed concentration of acetylcholine; *n* ≥ 1 independent experiment in triplicate.

**Scheme 1:**
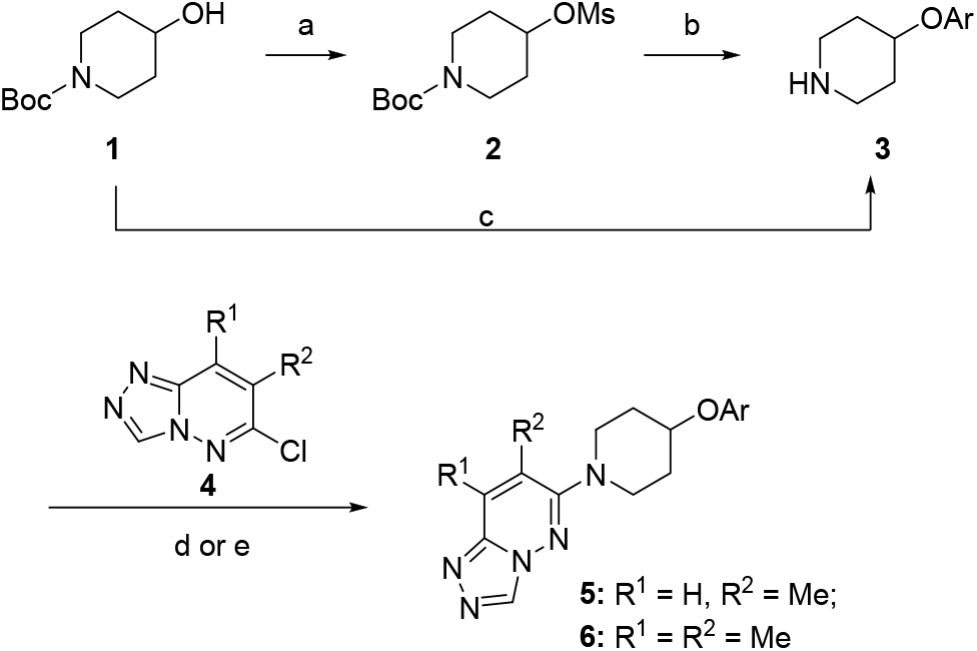
Synthesis of M_4_ PAM Aryl-Ethers **5** and **6** ^a^

a Reagents and conditions: (a) MsCl, TEA, DCM, 0 °C, 2 h, 99%; (b) i. ArOH, Bu_4_NCl, K_2_CO_3_, H_2_O, 100 °C; ii. TFA, DCM, 1–18 h, 10–86% (2 steps); (c) PPh_3_, DBAD, THF, 18 h, 8–77%; (d) **4**, DIEA, NMP, 175 °C, 18 h, 4–73%; (e) DIEA, MeCN, 85 °C, 4 d, 39–54%.

**Table 2:** *In Vitro* Rat and Human Hepatic Clearance, Human M_2_ Selectivity, and CYP Inhibition Data for Select Analogs

	**CL**_**hep**_ (mL/min/kg)				**CYP**_**450**_ **IC**_**50**_ **(μM)**			
**Cmpd.**	**Human**	**Rat**	**rM**_**4**_ **EC**_**50**_ **(nM) (%Ach)** ^a^	**hM**_**2**_ **EC**_**50**_ **(μM) (%Ach)** ^a^	**1A2**	**2C9**	**2D6**	**3A4**
**5l**	14	29	n.d.	Inactive	>30	3.6	4.4	9.3
**6l**	13	42	n.d.	Inactive	27.9	16.4	>30	5.1
**5m**	5.3	25	740 (96)	Inactive	>30	4.0	9.2	14.2
**6m**	10	29	271 (84)	Inactive	>30	>30	>30	12.8
**5q**	11	58	48 (93)	1.38 (52)	>30	6.1	>30	20.3
**6q**	7.3	46	95 (81)	Inactive	>30	>30	9.2	25.1
**29b**	2.4	55	231 (70)	0.67 (52)	>30	4.5	>30	9.3
**29c**	8.8	32	82 (73)	Inactive	>30	20.8	>30	16.7
**29d**	2.1	46	81 (80)	>10 (43)	>30	7.8	6.1	>30
**29e**	5.4	37	96 (85)	Inactive	>30	12.6	3.6	10.8
**29j**	5.8	41	100 (44)	4.00 (27)	>30	15.1	1.3	16.9
**33h**	8.5	40	70 (80)	2.99 (54)	>30	>30	2.3	11.4
**33i**	2.2	29	80 (68)	>10 (41)	>30	>30	2.9	20.4
**33j**	10	37	130 (85)	>10 (47)	27	>30	5.2	12.3
**33k**	7.5	11	92 (87)	>10 (33)	>30	>30	5.1	29
**33n**	9.1	42	188 (66)	Inactive	>30	>30	>30	29.5
**33o**	7.9	33	154 (72)	Inactive	>30	28.6	>30	20.7
**33p**	9.9	34	49 (73)	Inactive	>30	26.1	8.1	19.1
**33q**	2.1	34	62 (77)	Inactive	>30	>30	8.4	19.3
**33r**	0.36	43	55 (53)	1.32 (55)	>30	7.7	10.2	1.2
**39b**	9.9	44	233 (72)	5.59 (40)	>30	29.5	>30	>30
**39d**	12	51	214 (101)	Inactive	>30	5.9	>30	18.7
**39i**	1.7	29	289 (96)	Inactive	>30	3.1	4.0	4.7
**39n**	20	60	240 (59)	Inactive	>30	>30	>30	>30
**39r**	3.4	45	348 (96)	Inactive	>30	29.7	13.8	19.6

a Calcium mobilization assays with hM_4/Gqi5_-CHO cells performed in the presence of an EC_20_ fixed concentration of acetylcholine, *n* ≥ 1 independent experiment in triplicate. (n.d. = not determined).

To improve half-life, we devised a strategy for our third generation of analogs in which we modified the 2,3-dihydrobenzo­[*b*]­[1,4]­dioxine ring. These analogs were synthesized according to Schemes 2 and 3. We first began with the synthesis of the various 2,3-dihydrobenzo­[*b*]­[1,4]­dioxin-6-ols as highlighted in Scheme 2. Aldehyde **7** was first treated with 1,2-dibromoethane-1,1,2,2-*d*_4_, then subsequently oxidized with mCPBA to yield intermediate **8**. Intermediate **8** was further modified by first protecting the alcohol as the THP-ether followed by selective bromination using 1,2- dibromotetrafluoroethane and *n*-BuLi.^29^ Deprotection of the THP-ether yielded intermediate **9**. Starting diols **13** and **16** were likewise treated with 1,2-dibromoethane-1,1,2,2-*d*_4_ to yield intermediates **14** and **17**, respectively. Bromides **14** and **17** were then converted into their respective pinacol boranes which were further converted into alcohols **15** and **18** via oxidation-hydrolysis. Starting alcohol **10** was first alkylated with 1-bromopropan-2-ol then converted into intermediate **11** via an intramolecular Pd-catalyzed carbon–oxygen bond formation. Methyl ester **11** was then reduced with LAH to the benzyl alcohol followed by Dess-Martin oxidation to yield the corresponding aldehyde which was then converted into alcohol **12** in a similar manner as intermediate **8**. Final analogs could then be synthesized according to Scheme 3. With alcohols **8**, **12, 15**, and **18** in hand as well as commercially available 2,2,3,3-tetrafluoro-6-hydroxybenzodioxene, we could easily generate piperidines **20–24** via substitution followed by Boc-deprotection. Intermediate **23** (R^5^ = Me) was then purified by supercritical fluid chromatography (SFC) to yield enantiomerically pure material which was then carried forward. Alcohol **9** reacted with mesylate **19** to afford bromide **25**. Intermediate **25** then underwent either a Pd-catalyzed cyanation or Suzuki coupling reaction followed by Boc-deprotection to afford piperidines **26–28**. All piperidines reacted with chloride **4** to give the S_N_Ar products **29**. Analogs were screened against hM_4_ with results highlighted in Table 3.

**Scheme 2:**
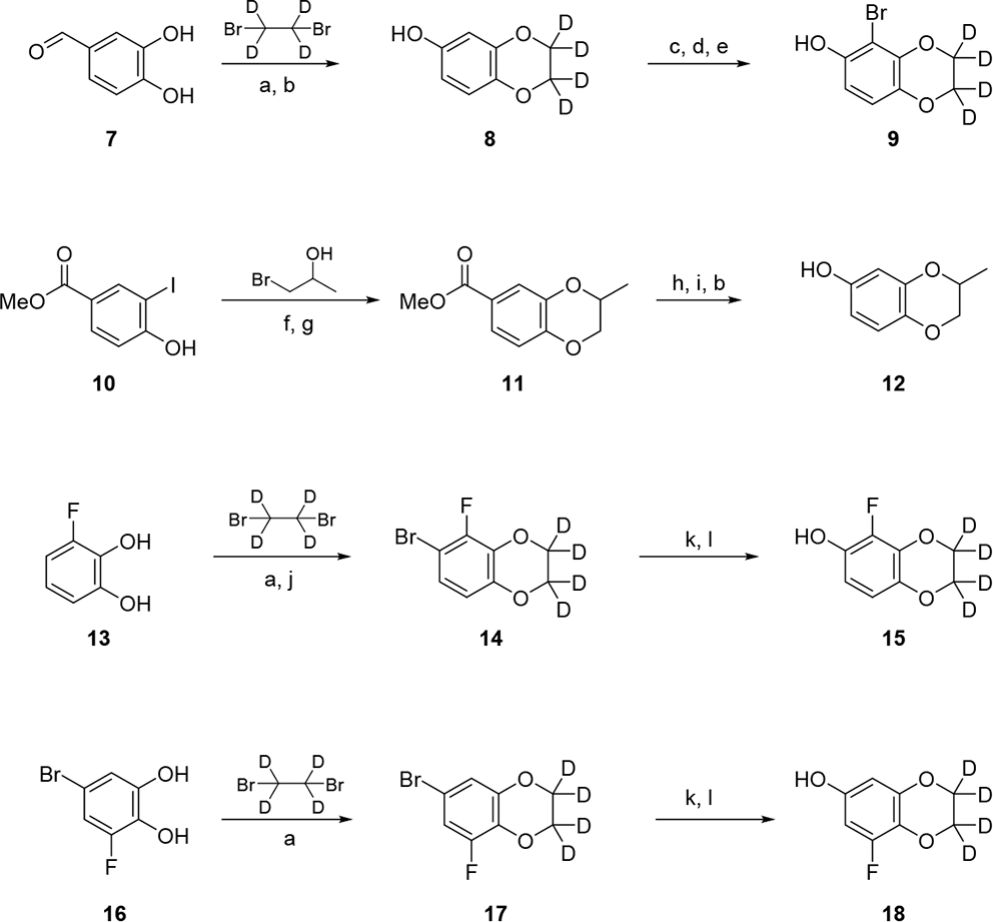
Synthesis of Modified 2,3-Dihydrobenzo­[*b*]­[1,4]­Dioxin-6-ol Intermediates^a^

a Reagents and conditions: (a) K_2_CO_3_, acetone, 60 °C, 18 h, 60%; (b) *m*CPBA, K_2_CO_3_, DCE, 50 °C, 18 h; then K_2_CO_3_, MeOH, rt, 3 h, 80%; (c) DHP, PPTS, DCM, rt, 80%; (d) 1,2-dibromotetrafluoroethane, *n-*BuLi, THF, −50 °C, 1 h, 82%; (e) HCl, THF, rt, 1 h, 93%; (f) KI, K_2_CO_3_, DMF, 80 °C, 72 h, 98%; (g) *t-*BuXPhos, Pd­(OAc)_2_, Cs_2_CO_3_, toluene, 110 °C, 18 h, 37%; (h) LAH, THF, 0 °C to rt, 1 h, 88%; (i) DMP, DCM, 4 h, rt, 61%; (j) Br_2_, NaOAc, chloroform, 0 °C to rt, 67%; (k) B_2_pin_2_, KOAc, Pd_2_(dppf)­Cl_2_·CH_2_Cl_2_, DMF, 90 °C, 18 h, 63%; (l) NaOH, H_2_O_2_, THF, 0 °C to rt, 1 h, 38%.

**Scheme 3:**
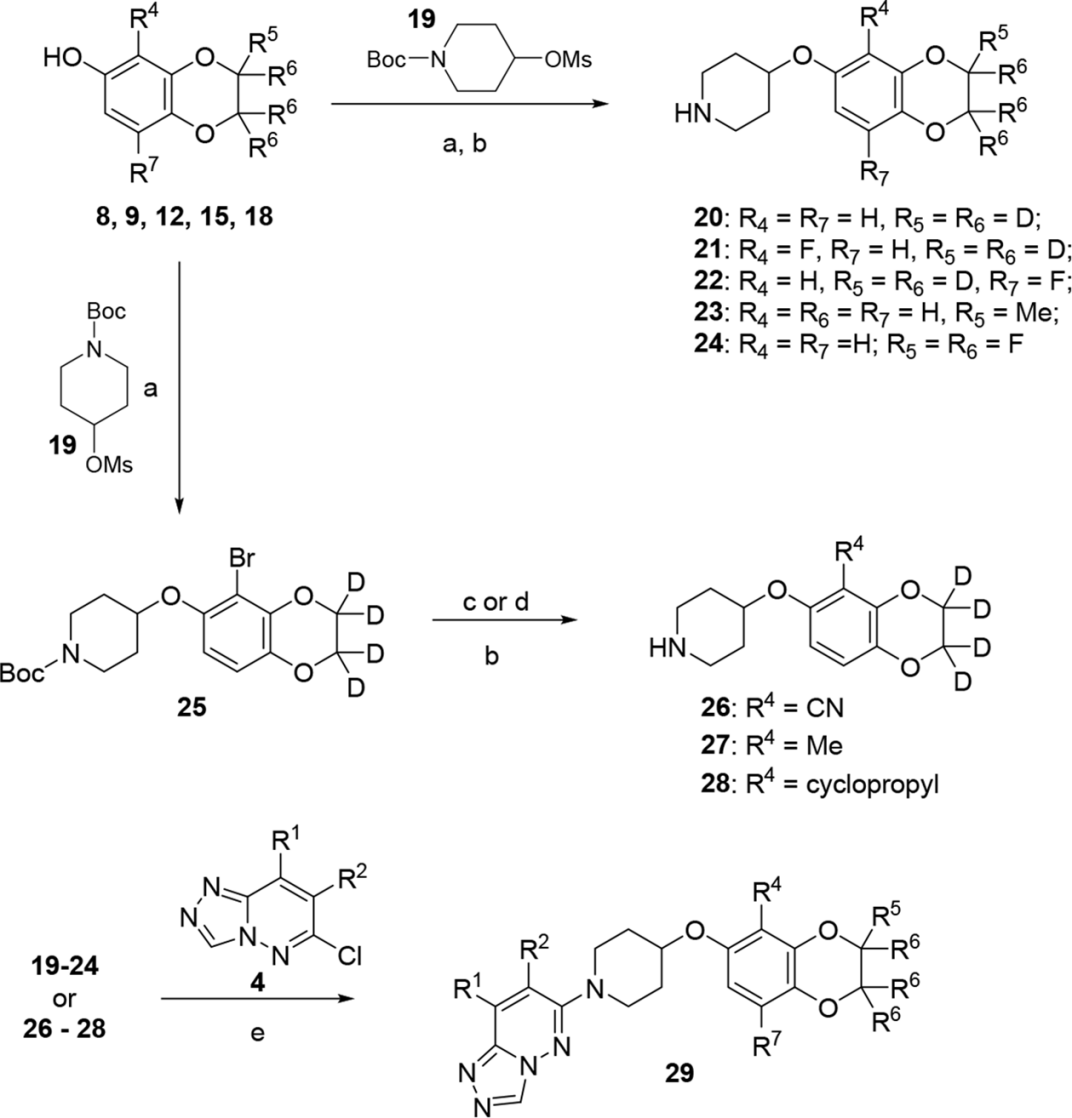
Synthesis of M_4_ PAM Analogs **29** ^a^

a Reagents and conditions: (a) **18**, Bu_4_NCl, K_2_CO_3_, DMF/H_2_O, 100 °C, 18 h, 40–60%; (b) TFA, DCM, rt, 1 h; 67 −85%; (c) trimethylboroxine or cyclopropyl boronic acid, Pd_2_(dppf)­Cl_2_·Cs_2_CO_3_, 1,4-dioxanes, 80 °C, 2 h, 36–47%; (d) Zn­(CN)_2_, Pd­(PPh_3_)_4_, DMF, microwave irradiated at 140 °C, 0.5 h, 77%; (e) DIEA, NMP, 175 °C, 18 h, 20–49%.

**Table 3:**
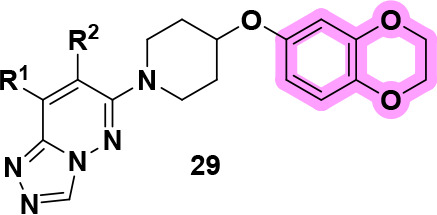

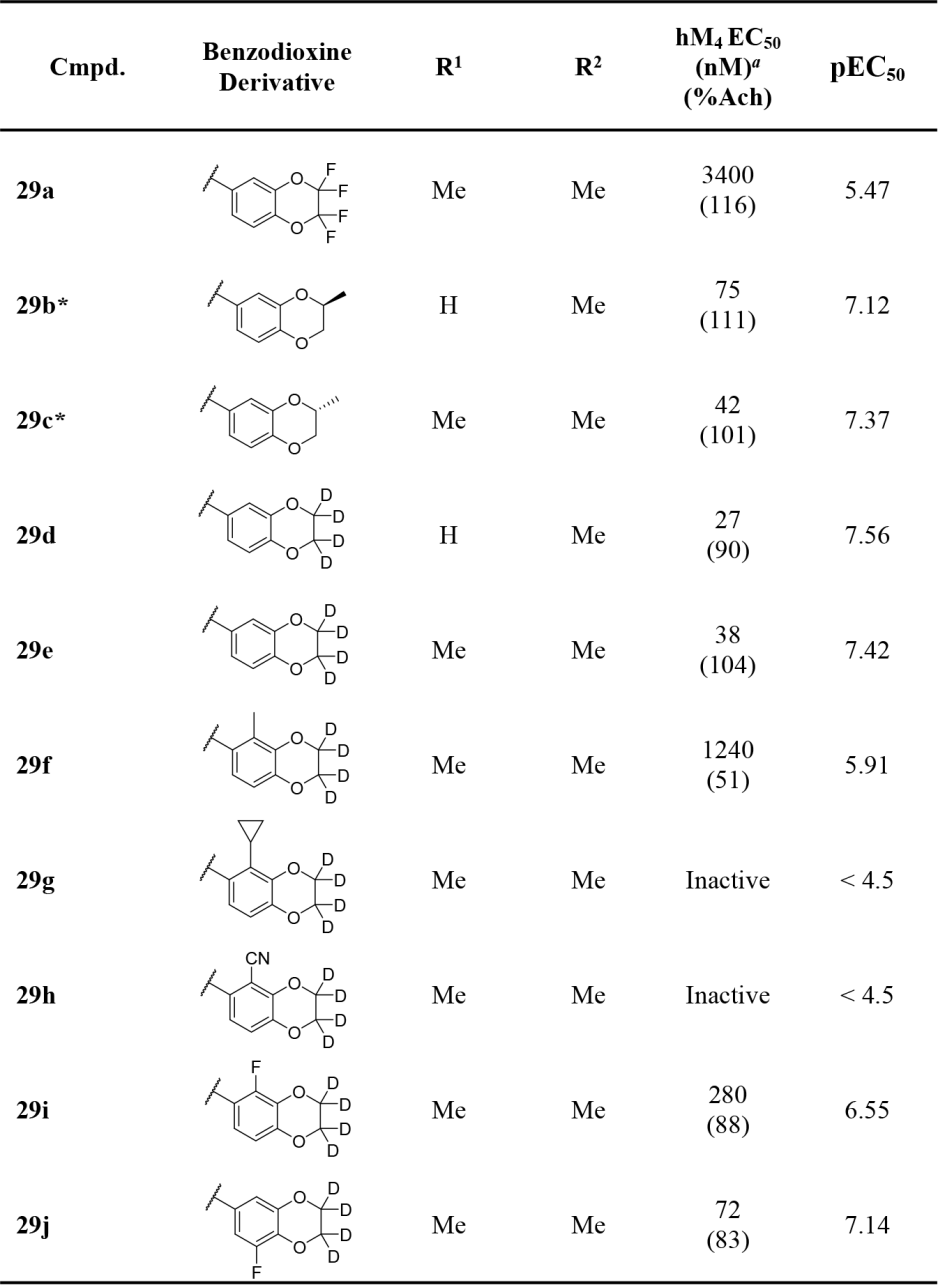
SAR of Third Generation M_4_ PAM Analogs 29^a^

a Calcium mobilization assays with hM4/Gqi5-CHO cells performed in the presence of an EC_20_ fixed concentration of acetylcholine; *n* ≥ 1 independent experiment in triplicate. *Single enantiomer of unknown absolute configuration.

Substituting the 2,3-dihydrobenzo­[*b*]­[1,4]­dioxine ring to give the tetrafluoro derivative **29a** resulted in a loss of potency (hM_4_ EC_50_ = 3.4 μM). The two 2-methyl-2,3-dihydrobenzo­[*b*]­[1,4]­dioxine enantiomers (**29b** and **29c**) provided analogs that were potent on both hM_4_ and rM_4_. Upon comparison, one enantiomer (**29b**: R^1^ = H) displayed low human CL_hep_ (2.4 mL/min/kg) but unfortunately exhibited hM_2_ activity (EC_50_ = 671 nM) (Table 2). Conversely, the other enantiomer (**29c**: R^1^ = Me) was inactive on hM_2_, but was determined to have a less desirable human CL_hep_ (8.8 mL/min/kg). It can be hypothesized that the loss of hM_2_ activity is due the presence of the additional methyl group on the [1,2,4]­triazolo­[4,3-*b*]­pyridazine ring (R^1^ = Me), as loss of hM_2_ activity is also observed with analog **29d** versus **29e**, in which the only point of difference is the additional 7-methyl group. We believe moderate human CL_hep_ can be attributed to the 2-methyl-2,3-dihydrobenzo­[*b*]­[1,4]­dioxine ring as opposed to the additional methyl on the [1,2,4]­triazolo­[4,3-*b*]­pyridazine ring. This is supported by the fact that the additional methyl did not prove unfavorable in regard to human CL_hep_ of compound **29e** versus **29d**. In fact, the additional methyl group on the [1,2,4]­triazolo­[4,3-*b*]­pyridazine ring of **6q** (hCL_hep_ = 7.3 mL/min/kg; rCL_hep_ = 30 mL/min/kg) versus **5q** (hCL_hep_ = 11 mL/min/kg; rCL_hep_ = 58 mL/min/kg) improved both rat and human predicted hepatic clearance values.

Replacement of the hydrogens on the 2,3-dihydrobenzo­[*b*]­[1,4]­dioxine ring with deuterium (**29d** and **29e**) did not greatly affect hM_4_ potencies when compared to **5q** and **6q**; however, this modification yielded compounds with low human predicted hepatic clearance (**29d**: CL_hep_ = 2.14 mL/min/kg; **29e**: CL_hep_ = 5.4 mL/min/kg), which was an improvement in comparison to **5q** and **6q**. More importantly, this modification improved the poor elimination half-life observed for **6q** (*t*_1/2_ = 0.6 h) to the more desirable half-life of **29e** (*t*_1/2_ = 8.8 h) but, unfortunately, we also observed increased CYP2D6 inhibition (IC_50_ = 3.6 μM). Further analysis revealed that substituting the aryl ring of the 2,3-dihydrobenzo­[*b*]­[1,4]­dioxine at the 5-position was detrimental to hM_4_ activity (**29f**, **29g**, **29h**, and **29i**). While fluorine was at least tolerated, larger groups at the 5-position greatly reduced potency and even afforded inactive compounds. Alternatively, substituting the aryl ring of the 2,3-dihydrobenzo­[*b*]­[1,4]­dioxine at the 8-position (**29j**) resulted in human M_4_ EC_50_’s < 100 nM as well as low human CL_hep_ (5.8 mL/min/kg) and moderate rat CL_hep_ (41 mL/min/kg). This modification, however, resulted in even greater CYP2D6 inhibition (IC_50_ = 1.3 μM).

With the improved half-life of **29e**, we turned our attention to rectifying the CYP inhibition profile. The fourth generation of analogs focused on alteration to the core piperidine ring (Table 4). Piperidine replacements **33a**–**33g** were synthesized in a straightforward manner from the commercially available Boc-protected amine alcohols in a similar manner as depicted in Scheme 1. Ring expansion of the piperidine (**33a** and **33b**) as well as ring contraction (**33c** and **33g**) afforded analogs that are either less potent than the parent piperidine compound (**5q** or **6q**) or inactive when screened against hM_4_. Piperidine bioisosteres **33e** and **33f** also proved to be significantly less potent than the original piperidine analog.

**Table 4:**
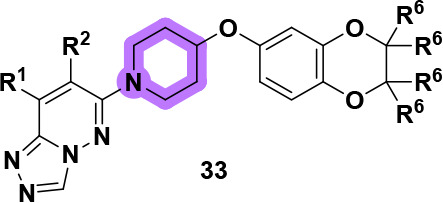

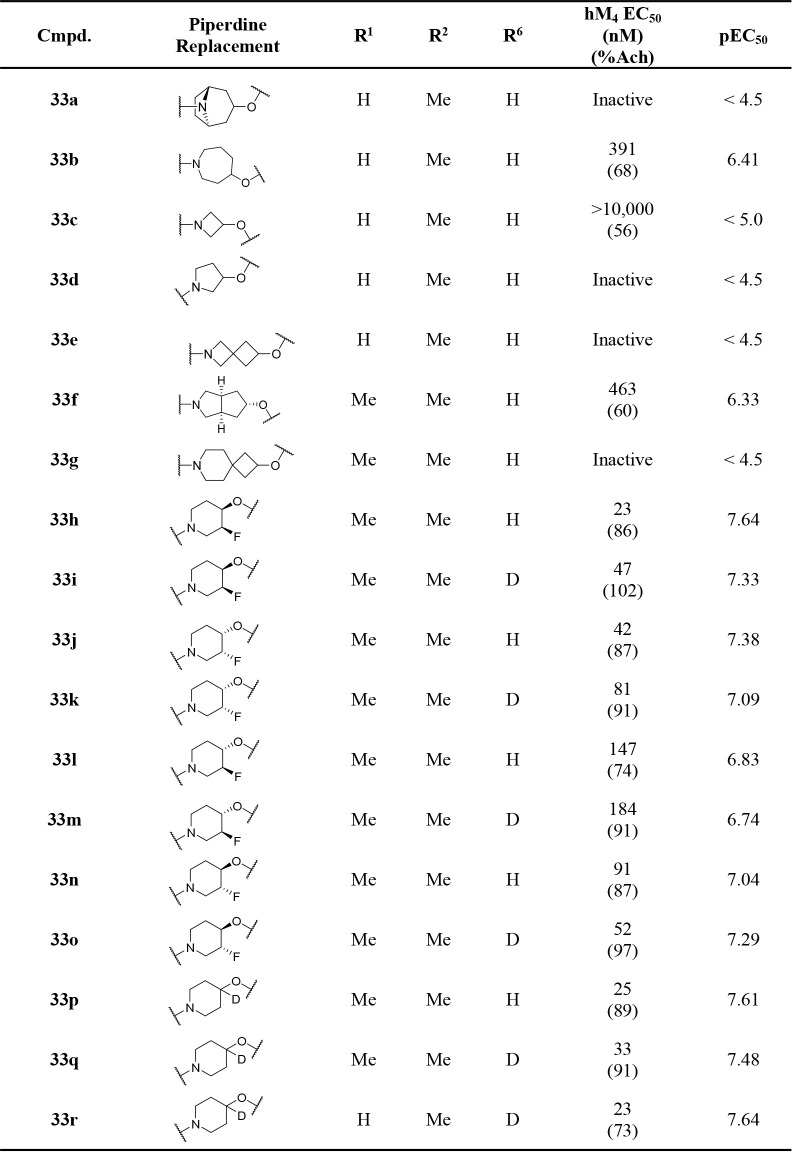
SAR of Fourth Generation M_4_ PAM Analogs 33^a^

a Calcium mobilization assays with hM_4/Gqi5_-CHO cells performed in the presence of an EC_20_ fixed concentration of acetylcholine; *n* ≥ 1 independent experiment in triplicate.

As deviation from the piperidine core was detrimental to the potency of our compounds, we focused our attention on generating substituted piperidine cores (**33h**–**33r**). Scheme 4 shows a representative synthetic route by which analogs **33h**–**33o** were all synthesized. In general, commercially available Boc-protected amines underwent a Mitsunobu reaction with phenol **8** or **8’** and, following Boc-deprotection, afforded amines **31** or **31’**. Following our standard S_N_Ar conditions with aryl chloride **32**, final compounds **33h–33o** were generated. Scheme 5 details a representative process to synthesized analogs **33p–33r**. In short, commercially available 1-boc-4-piperidone (**34**) was reduced with sodium borodeuteride. The resulting alcohol was then tosylated with TsCl to afford intermediate **35**. Following a substitution reaction with alcohol **8** or **8’** then subsequent Boc-deprotection yielded amines **36** or **36’**. This newly synthesized piperidine-4-*d* intermediate was then subjected to our standard S_N_Ar conditions with aryl chloride **32** to afford final compound **33p** and **33q**.

**Scheme 4:**
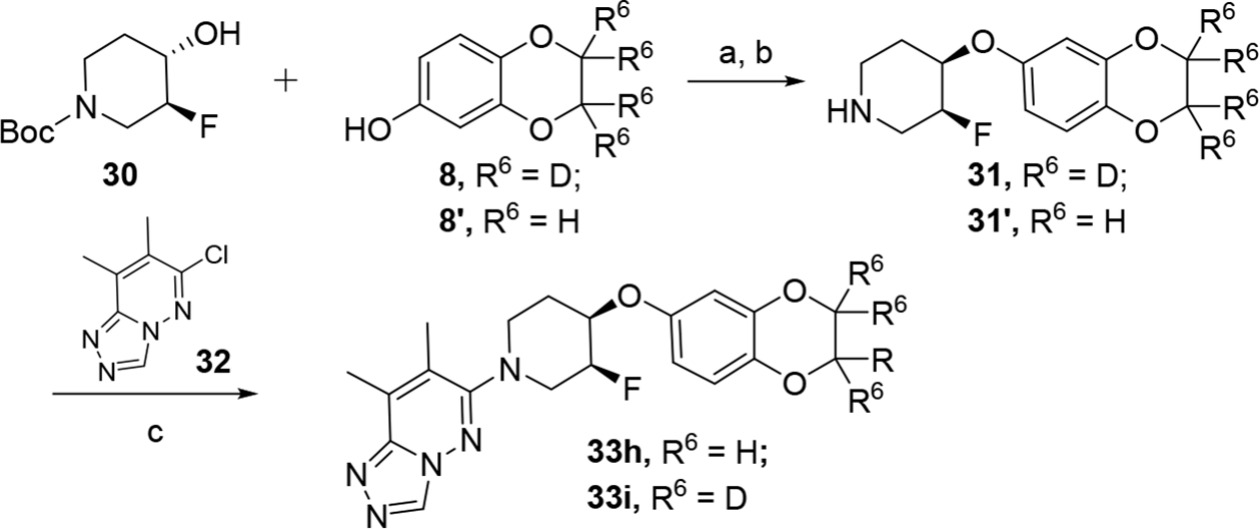
Synthesis of M_4_ PAM Analogs **33h** and **33i** ^a^

a Reagents and conditions: (a) ADDP, P­(*n*-Bu)_3_, toluene, 80 °C, 91%; (b) TFA, DCM, rt, 1h; 44%; (c) **32**, DIEA, NMP, 175 °C, 18 h, 56%.

**Scheme 5:**
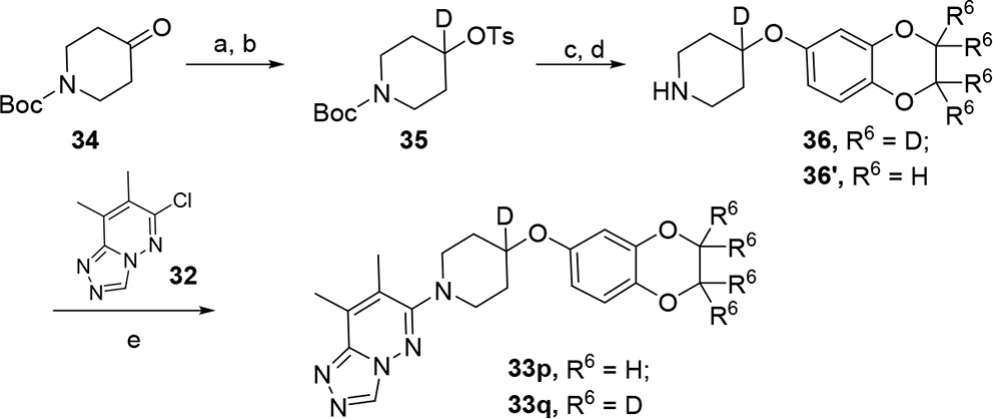
Synthesis of M_4_ PAM Analog **33p** and **33q** ^a^

a Reagents and conditions: (a) NaBD_4_, MeOH, 0 °C to rt, 4 h, 99%; (b) TsCl, DMAP, pyridine, 18 h, 82%; (c) **20**, Bu_4_NCl, K_2_CO_3_, DMF/H_2_O, 100 °C, 18 h; (d) TFA, DCM, rt, 1 h; 46% (2 steps); (e) **32**, DIEA, NMP, 175 °C, 18 h, 60%.

All fluoropiperidine analogs tested were highly potent against hM_4_ with EC_50_ *s* < 200 nM; however, the fluorinated (3*S*, 4*S*)-*trans* isomer (**33l** and **33m**) was the least favorable isomer in relation to hM_4_ potencies (EC_50_ > 140 nM) (Table 4). Conversely, the fluorinated (3*R*,4*R*)-*trans* isomers **33n** and **33o** were ∼2–4-fold more potent when screened for hM_4_ activity with moderate predicted human hepatic clearance (**33o**, CL_hep_ = 7.9 mL/min/kg) in comparison to its nonfluorinated counterpart which displayed low predicted human clearance (**29e**, CL_hep_ = 5.4 mL/min/kg) as shown in Table 2. By comparison, both fluorinated *cis*-isomers displayed ≤100 nM potencies against hM_4_. While the fluorinated (3*S*, 4*R*)-*cis* isomers **33h** and **33i** improved CYP2C9 inhibition (IC_50_’s = 30 μM) compared to **29e** (IC_50_ = 12.6 μM), both analogs suffered from an increase in CYP2D6 inhibition (**33h**: IC_50_ = 2.3 μM and **33i** IC_50_ = 2.9 μM) when compared to analog **29e** (IC_50_ = 3.6 μM). Additionally, the fluorinated (3*S*, 4*R*)-*cis* isomer resulted in a drastic decrease in the elimination half-life (**33i**, *t*_1/2_ = 0.83 h) when compared to **29e** (*t*_1/2_ = 8.8 h). Once again, the tetradeutero substitution on the 2,3-dihydrobenzo­[*b*]­[1,4]­dioxine ring displayed a trend of improved predicted hepatic clearance of **33i** (rCL_hep_ = 28.7 mL/min/kg; hCL_hep_ = 2.2 mL/min/kg) when compared to the nondeutero analog, **33h** (rCL_hep_ = 40.4 mL/min/kg; hCL_hep_ = 8.5 mL/min/kg). This trend was also observed with the (3*R*, 4*S*)-cis isomers **33k** rCL_hep_ = 11.1 mL/min/kg; hCL_hep_ = 7.5 mL/min/kg and **33j** (rCL_hep_ = 37 mL/min/kg; hCL_hep_ = 10 mL/min/kg). Moreover, the fluorinated (3*R*, 4*S*)-*cis* isomers **33j** and **33k** modestly improved CYP2D6 inhibition (**33j**: IC_50_ = 5.2 μM and **33k**: IC_50_ = 5.1 μM) when compared to analog **29e** (IC_50_ = 3.6 μM). Fluorination of the piperidine ring also improved predicted rat hepatic clearance (**33k**; rCL_hep_ = 11.1 mL/min/kg; hCL_hep_ = 7.5 mL/min/kg) when compared to the nonfluorinated analog **29e** (rCL_hep_ = 36.8 mL/min/kg; hCL_hep_ = 5.4 mL/min/kg) while having minimal effect on predicted human hepatic clearance. Moreover, the fluorinated (3*R*, 4*S*)-*cis* isomer also reduced the elimination half-life of our molecule (**33k**, *t*_1/2_ = 2.66 h), although to a lesser extent than the (3*S*, 4*R*)-*cis* isomer.

The most profound effect was noticed when a deuterium was incorporated into the piperidine ring (**33p** and **33q**; Tables 2 and 4). This modification not only improved CYP2D6 inhibition (**33p**: IC_50_ = 8.2 μM and **33q**: IC_50_ = 8.4 μM) but also CYP2C9 and CYP3A4 inhibition (IC_50_s > 19 μM) in comparison to **29e** (CYP2D6 IC_50_ = 3.6 μM; CYP2C9 IC_50_ = 12.6 μM; CYP3A4 IC_50_ = 10.8 μM) while maintaining hM_4_ (**33p**: EC_50_ = 25 nM **33q**: EC_50_ = 33 nM) and rat potency (**33p**: EC_50_ = 49 nM **33q**: EC_50_ = 62 nM). Once again, we noticed the tetradeutero substitution on the 2,3-dihydrobenzo­[*b*]­[1,4]­dioxine ring benefited human predicted hepatic clearance (**33q**, hCL_hep_ = 2.14 mL/min/kg) when compared to the nondeutero analog **33p** (hCL_hep_ = 9.9 mL/min/kg). Interestingly, we observed a less than desirable CYP profile upon deletion of the 7-methyl (R^1^) of the 7,8-dimethyl-[1,2,4]­triazolo­[4,3-*b*]­pyridazine headgroup (**33r**). The most profound effect was in relation to CYP3A4 (IC_50_ = 1.2 μM) as compared to the corresponding dimethyl analog **33q** (IC_50_ = 19.3 μM).

Finally, we shifted our focus toward modifications of the 7,8-dimethyl-[1,2,4]­triazolo­[4,3-*b*]­pyridazine headgroup to generate our fifth generation of analogs, **38** and **39**. To begin, we evaluated analogs containing historical head groups we have employed in the past when designing M_4_ PAMs.^30^ Heteroaryl bromides (**37**) underwent Buchwald-Hartwig aminations to afford analogs **38a-I** (Scheme 6). Disappointingly, as highlighted in Table 5, this approach proved unfruitful as these analogs were either inactive or showed micromolar activity when screened for activity against hM_4_. Undeterred by these results, we turned our attention to the synthesis of novel head groups that more closely resemble the original 7,8-dimethyl-[1,2,4]­triazolo­[4,3-*b*]­pyridazine headgroup or were direct modifications thereof. Briefly, heteroaryl chlorides **37** were subjected to standard S_N_Ar conditions to generate analogs **39** with results of this endeavor showcased in Table 6. The importance of the nitrogen at the 2-position of the [1,2,4]­triazolo­[4,3-*b*]­pyridazine ring was apparent as deletion of this nitrogen (**39a**) led to a 5.5-fold decrease in hM_4_ potency. Replacement of the original headgroup with a 2,7-dimethyl-[1,2,4]­triazolo­[1,5-*a*]­pyridine ring (**39b**) led to a slight decrease in potency in hM_4_ (2-fold). Additionally, this motif was detrimental to hCL_hep_ (9.9 mL/min/kg) and revealed hM_2_ activity (5.6 μM; Table 2). In general, substitution of the 7,8-dimethyl-[1,2,4]­triazolo­[4,3-*b*]­pyridazine ring at the 3-position (**39c – 39e**) resulted in a loss of hM_4_ potency. Interestingly, the difluoromethyl analog **39d** (hM_4_ EC_50_ = 78 nM) was ∼9-fold more potent than the corresponding trifluoromethyl analog **39c** (hM_4_ EC_50_ = 699 nM); however, **39d** was still over 2-fold less potent than the parent compound **33q** (hM_4_ EC_50_ = 35 nM). Moreover, the difluoromethyl substitution had an undesirable effect on the hCL_hep_ (12 mL/min/kg).

**Table 5:**
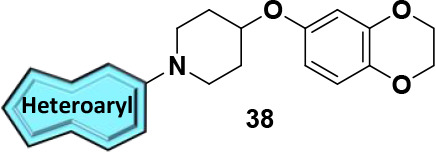

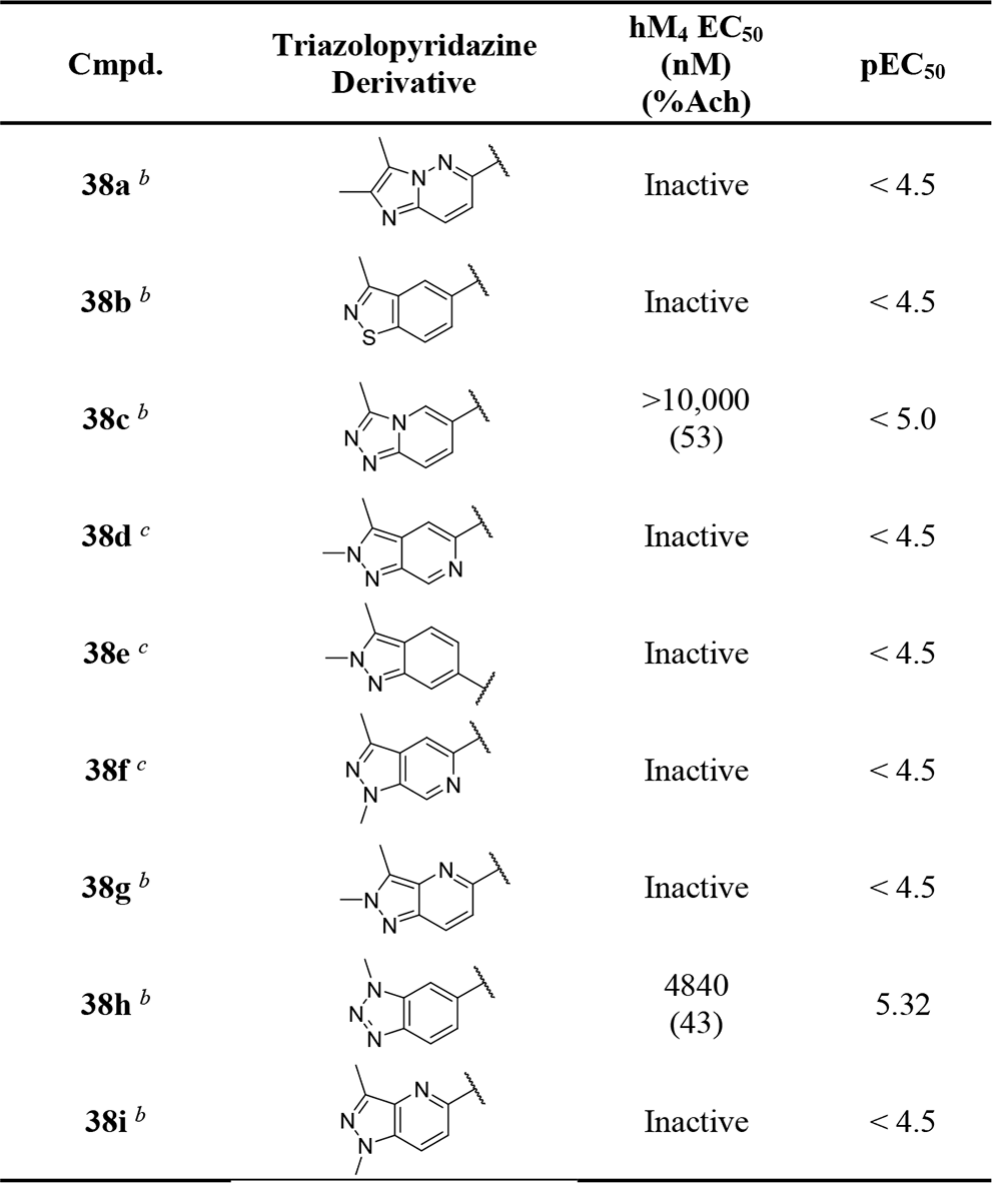
SAR of Analogs **38** Containing Historical M_4_ PAM Head Groups^a^

a Calcium mobilization assays with hM_4/Gqi5_-CHO cells performed in the presence of an EC_20_ fixed concentration of acetylcholine, *n* = 1 in triplicate. ^*b*^ Synthesized according to Scheme 6, condition a; ^*c*^ Synthesized according to Scheme 6, condition b.

**Scheme 6:**
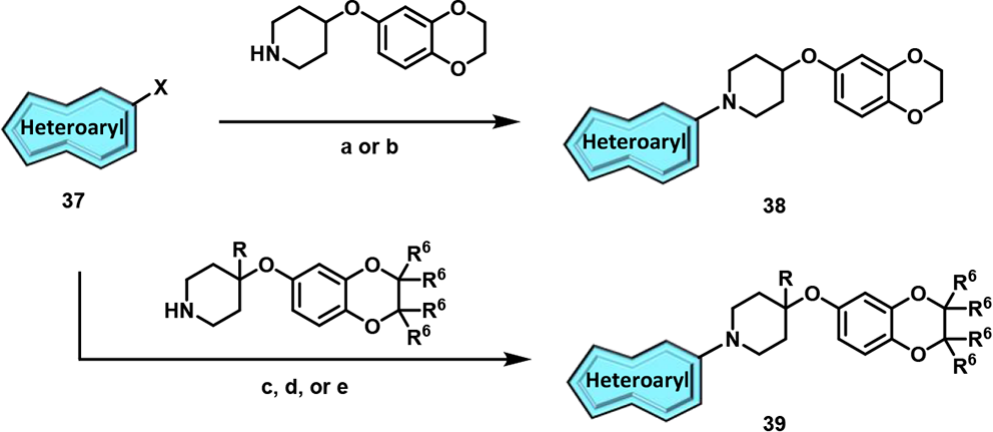
Synthesis of M_4_ PAM Analogs **38** and **39** ^a^

a Reagents and conditions: (a) Pd_2_(dba)_3_, Xantphos, Cs_2_CO_3_, 1,4-dioxanes, 100 °C, 13–90%; (b) ^*t*^BuXPhos Pd G1, ^*t*^BuXPhos, ^*t*^BuONa, ^*t*^BuOH, 50 °C (16–41%); (c) DIEA, NMP, 175 °C, 10–95%; (d) Pd_2_(dba)_3_, rac-BINAP, ^*t*^BuONa, toluene, 120 °C, 12%; (e) Pd_2_(dba)_3_, Xantphos, RuPhos Pd G3, Cs_2_CO_3_, 1,4-dioxanes, 100 °C, 33%.

**Table 6:**
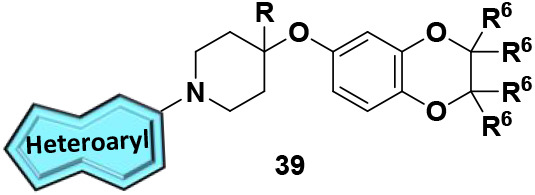

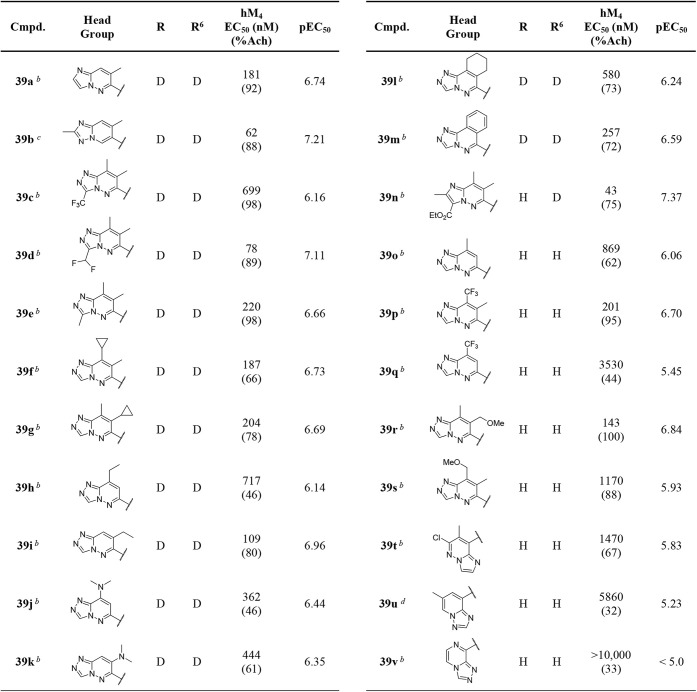
SAR of Fifth Generation M_4_ PAM Analogs 39^a^

a Calcium mobilization assays with hM_4/Gqi5_-CHO cells performed in the presence of an EC_20_ fixed concentration of acetylcholine; *n* ≥ 1 independent experiment in triplicate. ^b^Synthesized according to Scheme 6, condition c; ^c^synthesized according to Scheme 6, condition d; ^d^synthesized according to Scheme 6, condition e.

Varying the substitutions at the 7 or 8-positions of the [1,2,4]­triazolo­[4,3-*b*]­pyridazine ring consistently produced less active compounds (**39f–39m** and **39o–39s**). The importance of the 8-methyl (R^2^) of the 7,8-dimethyl-[1,2,4]­triazolo­[4,3-*b*]­pyridazine ring was demonstrated by 7-methyl analog **39o** (hM_4_ EC_50_ = 869 nM) which was 6.5-fold less potent when compared to the 8-methyl analog **5q** (hM_4_ EC_50_ = 134 nM) and 23-fold less potent when compared to the 7,8-dimethyl analog **6g** (hM_4_ EC_50_ = 38 nM). More surprising was the ∼23-fold loss in activity when **39o** was compared to the corresponding 7,8-dimethyl analog **6q** (hM_4_ EC_50_ = 38 nM). Intriguingly, introducing larger groups at the 7-position, such as an ethyl (**39h**, hM_4_ EC_50_ = 717 nM) or diethylamine (**39j**, hM_4_ EC_50_ = 362 nM), resulted in a 1.2–2.4-fold increase in hM_4_ activity, respectively, when compared to **39o**. We postulate that bulkier groups at the 7-position are extending into a hydrophobic pocket originally occupied by the 8-methyl group of the 7,8-dimethyl-[1,2,4]­triazolo­[4,3-*b*]­pyridazine headgroup. This trend is also observed when comparing analogs **39p** and **39q**; the presence of the 8-methyl group yields a 17.6-fold more active analog. Replacing the 8-methyl group of analog **33r** (hM_4_ EC_50_ = 23 nM) with a larger ethyl group yielded analog **39i** (hM_4_ EC_50_ = 109 nM). Although **39i** is ∼4.7-fold less potent than **33r**, it still displayed low nanomolar activity; however, further evaluation revealed that this modification was a detriment to the CYP profile (CYP2C9 IC_50_ = 3.1 μM; CYP2D6 IC_50_ = 4 μM; CYP3A4 IC_50_ = 4.7 μM) as shown in Table 2.

A more in-depth investigation into varying the substitutions at the 7 and 8-positions of the [1,2,4]­triazolo­[4,3-*b*]­pyridazine ring revealed a loss in potency of all analogs tested (**39f**, **39g**, **39p**, **39r**, and **39s**). Simply substituting the 7-methyl group of **6q** with a trifluoromethyl-group to yield **39p** led to a 5.3-fold decrease in hM_4_ activity. When compared to parent analog **33q** (hM_4_ EC_50_ = 33 nM), the cyclopropyl derivatives **39f** and **39g** also exhibited 5.7-fold and 6.2-fold losses in hM_4_ activity, respectively. Likewise, analogs **39r** and **39s** displayed a 3.8-fold and 31-fold loss in hM_4_ activity, respectively, when compared to parent analog **6q** (hM_4_ EC_50_ = 38 nM). While the 7-cyclopropyl analog (**39f**) and the 8-cyclopropyl analog (**39g**) were nearly equipotent to one another, the 8-methoxymethyl derivative (**39r**) was nearly 8.2-fold more potent than the 7-methoxymethyl compound (**39s**). Attempts to “tie-back” the 7-methyl and 8-methyl into a tricyclic ring system (**39l** and **39m**) also resulted in a loss of hM_4_ potency. Additionally, deviation from the [1,2,4]­triazolo­[4,3-*b*]­pyridazine core, in general, afforded analogs displaying a loss of hM_4_ activity (**39t**, **39u**, and **39v**) with the exception of **39n** (hM_4_ EC_50_ = 43 nM). Although the most active analog of our fifth generation series, compound **39n** suffered from a human-rat M_4_ potency discrepancy (4.4-fold, rM_4_ EC_50_ = 188 nM).

### Molecular Pharmacology and DMPK Profiling

Using the data summarized in Table 2, we rapidly deprioritized compounds with moderate to high predicted hepatic clearance (rat or human), potency discrepancies between species, rat potency (rM_4_ EC_50_ ≥ 200 nM), lack of hM_2_ selectivity, and/or less desirable CYP450 inhibition. As a result, only one compound stood out as a compound of interest for further profiling; thus, compound **33q** (**VU6025733**) was carried forward and evaluated for muscarinic selectivity as well as further *in vitro* and *in vivo* DMPK profiling (Table 7). Regarding physicochemical properties, **VU6025733** possesses an attractive molecular weight of <400 Da as well as a desirable CNS xLogP (2.99) and tPSA (74 Å^2^). When screened against other subtypes of muscarinic acetylcholine receptors (M_1_, M_2_, M_3_, and M_5_), **VU6025733** displayed high receptor subtype selectivity as it was inactive on both the human and rat isoforms of all other subtypes. Moreover, **VU6025733** exhibited no appreciable species differences in M_4_ activity between human and rat (∼2-fold) (Tables 2 and 4). **VU6025733** demonstrated acceptable CYP_450_ profiles against CYP1A2, CYP2C9, and CYP3A4 (IC_50_s ≥ 19.3 μM, > 500-fold selectivity) as well as CYP2D (IC_50_s = 8.4 μM, 240-fold selectivity). **VU6025733** displayed low human (CL_hep_ = 2.14 mL/min/kg) and moderate rat (CL_hep_ = 34 mL/min/kg) hepatic clearance based on microsomal intrinsic clearance (CL_int_). **VU6025733** had moderate fraction unbound in rat and human plasma (*f*_u,plasma_ = 0.010 and *f*_u,plasma_ = 0.051, respectively) and moderate rat brain homogenate binding (*f*_u,brain_ = 0.016). Next, we assessed *in vitro* brain

**Table 7:** Muscarinic Selectivity Data and DMPK Analysis for **VU6025733** (**33q**)­^a^

**Property**	**33q VU6025733**
MW (g/mol)	386.5
xLogP	2.99
TPSA (Å2)	74
Muscarinic selectivity^a^	
Human M_1_, M_2_, M_3_, M_5_	Inactive
Rat M_1_, M_2_, M_3_, M_5_	Inactive
***In vitro*** **PK parameters**	
CL_int_ (mL/min/kg), rat	66
CL_hep_ (mL/min/kg), rat	34
CL_int_ (mL/min/kg), human	2.3
CL_hep_ (mL/min/kg), human	2.1
Rat *f*_u,plasma_ ^b^	0.010
Human *f*_u,plasma_ ^b^	0.051
Rat *f*_u,brain_ ^b^	0.016
**Brain distribution (0.25 h) (SD Rat; 0.2** mg/kg **IV)**	
*K*_p, brain:plasma_ ^c^	0.39
*K*_p,uu, brain:plasma_ ^d^	0.78
Rat IV PK^e^	
*t*_1/2_ (hr)	5.67
MRT (hr)	3.83
CL_p_ (mL/min/kg)	5.26
V_ss_ (L/kg)	1.21
Rat PO PK^f^	
*T*_max_ (hr)	0.75
*C*_max_ (ng/mL)	1,783
AUC_0‑∞_ (hr·ng/mL)	6,753
%F	74.1

a Calcium mobilization assay; values are an *n* ≥ 1 independent experiments in triplicate.

b *f*_u_ = Fraction unbound; equilibrium dialysis assay; brain = rat brain homogenates.

c *K*_p_ = total brain to total plasma ratio.

d *K*_p,uu_ = unbound brain (brain *f*_u_ × total brain) to unbound plasma (plasma *f*_u_ × total plasma) ratio.

e Male Sprague–Dawley rats (*n* = 2); vehicle = 10% EtOH, 40% PEG 400, 50% saline (1 mL/kg); dose = 1 mg/kg.

f Male Sprague–Dawley rats (*n* = 2); vehicle = 0.1% Tween-80, 0.5% methyl cellulose, 99.4% water (10 mL/kg); dose = 3 mg/kg.

penetration potential utilizing MDCKII-MDR1 transfected cells. **VU6025733** exhibited an efflux ratio (ER) of 0.80 and a *P*_app_ (A-B) of 12.7 × 10^–6^ indicating high brain penetration and lack of P-glycoprotein 1 (P-gp) efflux transport. Furthermore, **VU6025733** was administered in a rat PBL IV cassette study to determine the plasma/brain partition ratio. This analysis revealed our candidate showed a *K*_p_ = 0.39 and a *K*_p,uu_ = 0.78. Additionally, our candidate showed low plasma clearance in rat (CL_p_ = 5.26 mL/min/kg) with an acceptable volume of distribution (*V*_ss_ = 1.21 L/kg), and a desirable half-life of 4.8 h. When **VU6025733** was administered to rats at a PO dose of 3 mg/kg, our candidate displayed moderate to high oral bioavailability (%F = 74.1) with rapid absorption and low interanimal variability.

### Behavioral Pharmacology

With **VU6025733** in hand, we evaluated this compound in a preclinical model of antipsychotic-like activity utilizing **VU0467154** as a positive comparator.^26,31^ **VU6025733** demonstrated a robust dose-dependent blockade of amphetamine-induced hyperlocomotion (AHL) after oral administration following a 30 min pretreatment interval in rats (MED = 10 mg/kg, Figure 3). At the end of the study, brain:plasma K_p_s and K_p,uu_s were determined at all dose groups (K_p_s = 0.25–0.35; K_p,uu_s = 0.39–0.44) with mean *C*_brain,unbound_ ranging from 17.4 ng/g (10 mg/kg) to 50.5 ng/g (30 mg/kg) (Table 8). These data were in alignment with our PBL cassette data. Given the promising profile of **VU6025733** thus far, the compound was progressed toward a battery of genotoxicity and multiparametric cytotoxicity assays.

**Figure 3:**
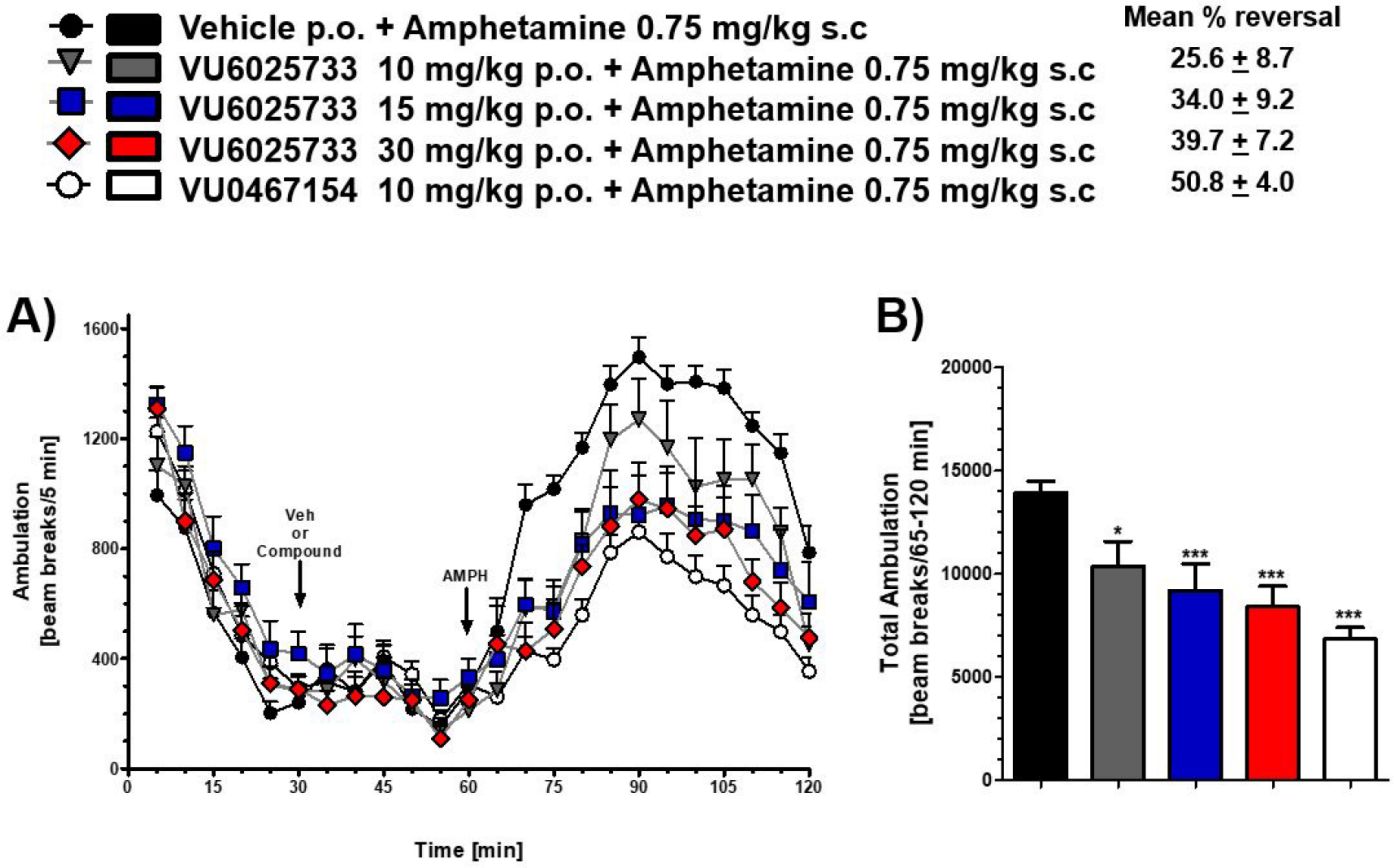
Systemic PO administration of **VU6025733 (33q)** blocked amphetamine-induced hyperlocomotion in male Sprague–Dawley rats. (A) The time course of locomotor activity and (B) Total locomotor activity during the 55 min period following amphetamine administration. Data are means ± SEM of 7–8 animals per group. *p < 0.05, **p < 0.01, ***p < 0.001 vs Vehicle + Amphetamine. Vehicle = 10% Tween 80 in H_2_O. **VU0467154** is a positive control.

**Table 8:** Relationship between Total (Mean *C*_brain_) and Unbound (Mean *C*_brain,u_) Brain Concentrations of **VU6025733** (**33q**) and Pharmacodynamic Effects on Amphetamine (0.75 mg/kg , SC)-Induced Hyperlocomotion in Rats at 1.5 h

**Dose** **(mg/kg)**	**Mean reversal of AHL (%)**	**Mean** ***C***_plasma_ ^a^ (ng/mL)	**Mean** ***C***_plasma,u_ ^b^ (ng/mL)	**Mean** ***C***_brain_ ^a^ (ng/g)	**Mean** ***C***_brain,u_ ^c^ (ng/g)	Brain:**p**lasma mean ***K***_**p**_	Brain:**p**lasma **m**ean ***K***_**p,uu**_
10	25.6	3,974	39.7	1,086	17.4	0.27	0.44
15	34.0	6,463	64.6	1,587	25.4	0.25	0.39
30	39.7	11,477	115	3,157	50.5	0.28	0.44

a At 1.5 h postadministration.

b Estimated unbound plasma concentration based on the rat *f*_u,plasma_ (0.010).

c Estimated unbound brain concentration based on the rat *f*_u,brain_ (0.016).

### Cytotoxicity and Toxicology Profile

With **VU6025733** displaying an attractive profile thus far, attention turned to assessing its viability as a development candidate. To assess potential cardiotoxicity, **VU6025733** was evaluated in a hERG SyncroPatch assay and was determined to have an IC_50_ of 4.6 μM, which was considered concerning, as human exposure projections were 1.06 μM, providing a narrow 4.4x margin. In genotoxicity assays, **VU6025733** was negative in both AMES (5 strain with and without S9) as well as negative in the *in vitro* micronucleus assay. PAM **VU6025733** was then advanced into a multiparametric cytotoxicity assay (Figure 4) in HepaRG 3D spheroids. Here, **VU6025733** had a very concerning profile, decreasing spheroid size, increasing oxidative stress, decreasing glutathione content (MEC = 9.7 μM, AC_50_ = 39 μM) and decreasing cellular ATP (MEC = 4.2 μM, AC_50_ = 6.0 μM). A follow-up evaluation of **VU6025733** in HepG2 cells demonstrated a decrease in oxygen consumption rate (MEC = 2.0 μM) and an increase in extracellular acidification rate (MEC = 7.1 μM). Combined, these data indicate that **VU6025733** is an electron transport chain inhibitor with only a ∼2-fold margin of the human exposure projection. Thus, there is predicted to be a very high risk of hepatotoxic side effects for **VU6025733**. Coupled with the narrow hERG margin, development of **VU6025733** was terminated.

**Figure 4:**
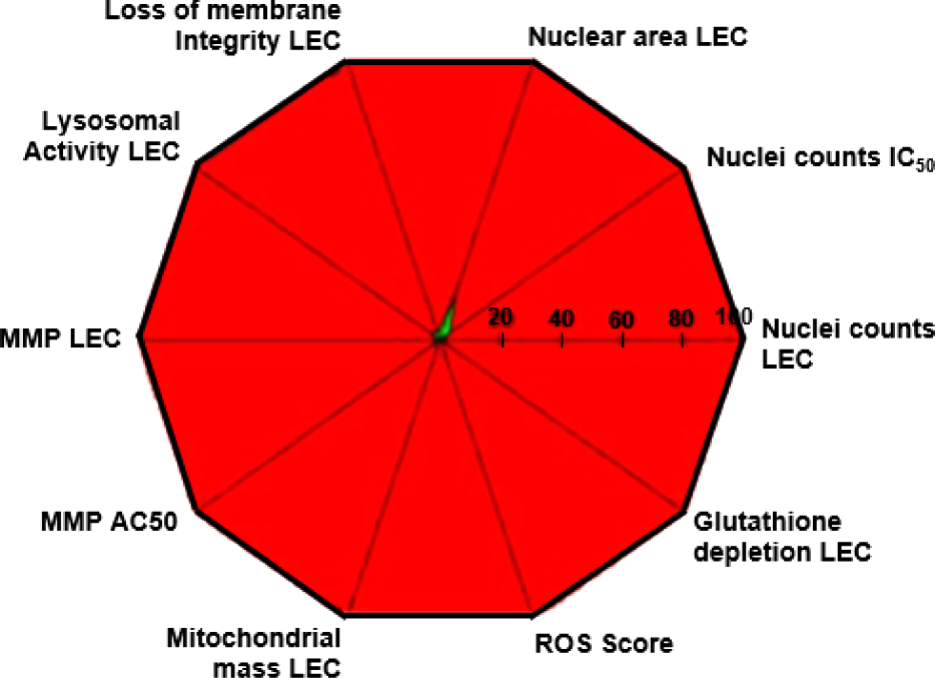
Multiparametric cytotoxicity in HepG2 cells obtained by high content imaging using 4 separate fluorescent and/or potentiometric probes. PAM **VU6025733** (**33q**) was broadly cytotoxic and proven to be an electron transport chain inhibitor (MEC ∼ 2 mM) with less than a 2-fold margin for human exposure projection. The graph shows a concentration range on its radials, from 0 (center) to 100 μM (edge). The lower the “lowest effective concentrations” (LEC) for each measured parameter, the larger the red area of the graph will be (contrary to the green center), suggesting an unfavorable safety profile for **VU6025733**. MMP = mitochondrial membrane potential.

## Conclusions

In summary, hybridizing the chemical scaffolds of previously disclosed M_4_ PAMs with unique scaffolds identified via an HTS (**VU0641491** and **VU0641483**, Figure 1) resulted in the discovery of novel PAMs **VU6015863** and **VU6020378**. Further medicinal chemistry efforts identified several highly potent (hM_4_ EC_50_ < 100 nM) M_4_ PAM analogs. Of these, analog **VU6025733** (**33q**) provided a superior overall profile that supported further progression. **VU6025733** not only displayed high selectivity over the other mAChRs evaluated (M_1–3_,_5_) but also demonstrated M_4_ potency agreement between species (human and rat). Moreover, **VU6025733** exhibited a low predicted hepatic clearance profile in human as well as low *in vivo* plasma clearance in rat. This was a considerable improvement over lead compounds **VU6015863** (hCL_hep_ = 11 mL/min/kg) and **VU6020378** (hCL_hep_ = 16 mL/min/kg). **VU6025733** displayed moderate to high CNS distribution of unbound drug (*K*_p,uu_ = 0.78) as well as modest brain and plasma fraction unbound in rat. Not only was **VU6025733** highly brain penetrant and not a substrate for the efflux transporter P-gp (ER = 0.80; a *P*_app_ (A-B) = 12.7 × 10^–6^) but the compound also demonstrated an acceptable CYP inhibition profile with IC_50_s ≥ 8.4 μM. Due to its attractive DMPK profile, **VU6025733** was advanced into *in vivo* pharmacokinetic/pharmacodynamic (PK/PD) profiling. **VU6025733** showed robust efficacy in a preclinical model of antipsychotic activity (AHL) after oral administration with an MED of 15 mg/kg. It is important to note that due to other setbacks, nonmuscarinic off-target activity, such as dopaminergic modulation, was not assessed during this study.

Finally, we evaluated the cytotoxicity and toxicology profile of **VU6025733**. Although **VU6025733** was negative in genotoxicity assays (AMES and *in vitro* micronucleus), further profiling revealed a narrow safety margin in relation to hERG inhibition. Advancement into a multiparametric cytotoxicity assay indicated a **VU6025733** is an electron transport chain inhibitor likely to have a very high risk of hepatotoxic side effects. For these reasons, further development of **VU6025733** was discontinued. Subsequent efforts suggest the [1,2,4]­triazolo­[4,3-*b*]­pyridazine headgroup as the key toxicophore, details of which will be provided in due course.

## Methods

### General Information^32^

All chemicals were purchased from commercial vendors and used without further purification. All NMR spectra were recorded on a 400 MHz AMX Bruker NMR spectrometer. ^1^H and ^13^C chemical shifts are reported in δ values in ppm downfield with the deuterated solvent as the internal standard. Low resolution mass spectra were obtained on an Agilent 6120/6150 or Waters QDa (Performance) SQ MS with ESI source. High resolution mass spectra were obtained on an Agilent 6540 UHD Q-TOF with ESI source. Normal phase column chromatography was performed on a Teledyne ISCO CombiFlash Rf+ system. For compounds that were purified on a Gilson preparative reversed-phase HPLC, the system comprised of a 333 aqueous pump with solvent selection valve, 334 organic pump, GX 271 or GX-281 liquid hander, two column switching valves, and a 155 UV detector. Solvents for extraction, washing and chromatography were HPLC grade. All final compounds were found to be >95% pure by HPLC-MS analysis.

### Synthesis^32^

#### Synthesis of 6-Chloro-7,8-dimethyl-[1,2,4]­Triazolo­[4,3-*b*]­pyridazine (**32**)

3,6-Dichloro-4,5-dimethylpyridazine (1.0 g, 5.6 mmol) and potassium carbonate (79 mg, 0.56 mmol) were dissolved in THF (28 mL) before the addition of hydrazine (890 μL, 28.2 mmol) dropwise under N_2_ atmosphere. The reaction mixture was heated to reflux. After 72 h, the reaction was concentrated *in vacuo* and used without further purification (975 mg). LRMS: C_6_H_9_ClN_4_ [M + H]^+^ calc. mass 173.0, found 173.3. The crude residue of 3-chloro-6-hydrazineylidene-4,5-dimethyl-1,6-dihydropyridazine (975 mg, 5.6 mmol) and formic acid (1.06 mL) were added to a sealed vessel. After heating at 100 °C for 1 h, the mixture was concentrated *in vacuo*. The crude material was dissolved in DCM, washed with 10% aqueous K_2_CO_3_ and back extracted with DCM (2×). The combined organic layers were dried (MgSO_4_), filtered, and concentrated in vacuo. The crude material was purified using flash chromatography on silica gel to afford the title compound (755 mg). ^1^H NMR (400 MHz, CDCl_3_) δ 8.89 (s, 1H), 2.67 (s, 3H), 2.36 (s, 3H). LRMS: C_7_H_7_ClN_4_ [M + H]^+^ calc. mass 183.0, found 183.4.

#### *tert*-Butyl 4-(Tosyloxy)­piperidine-1-carboxylate-4-*d* **(35)**

To a 0 °C solution of 1-*tert*-butyl-4-piperidone (10 g, 50 mmol) in methanol (250 mL) was added sodium borodeuteride (3.2 mL, 100 mmol). The resulting mixture was stirred for 4 h at room temperature. The reaction was quenched with saturated NH_4_Cl and extracted with EtOAc (3x). The combined organic layers were dried (MgSO_4_), filtered, and concentrated. To a suspension the crude residue and 4-dimethylaminopyridine (0.6 g, 4.9 mmol) in pyridine (45 mL) was added tosyl chloride (11.8 g, 62 mmol). The mixture stirred at room temperature for 18 h. The reaction was quenched with saturated aqueous NaHCO_3_ solution and extracted with EtOAc (2×). The combined organic layers were washed with water (2×), brine (2×), dried (MgSO_4_), filtered, and concentrated. The crude oil was purified via normal-phase chromatography on silica gel (0–20% EtOAc/Hexanes) to provide the title compound (14.3 g). ^1^H NMR (400 MHz, CDCl_3_) δ 7.79 (d, *J* = 8.4 Hz, 2H), 7.34 (d, *J* = 8.0 Hz, 2H), 3.61–3.55 (m, 2H), 3.28–3.22 (m, 2H), 2.45 (s, 3H), 1.79–1.73 (m, 2H), 1.70–1.64 (m, 2H), 1.43 (s, 9H). LRMS: C_17_H_24_DNO_5_S [M + Na]^+^ calc. mass 379.1, found 379.4.

#### 4-((2,3-Dihydrobenzo­[b]­[1,4]­dioxin-6-yl-2,2,3,3-*d*_4_)­oxy)­piperidine-4-*d* **(36)**

To a round-bottom flask were added 2,2,3,3-tetradeuterio-1,4-benzodioxin-6-ol (1.0 g, 6.7 mmol), *tert*-butyl 4-deuterio-4-(p-tolylsulfonyloxy)­piperidine-1-carboxylate (2.0 g, 5.6 mmol), potassium carbonate (2.4 g, 16.8 mmol), and tetrabutylammonium chloride (0.31 g, 1.1 mmol) in water (25 mL) and DMF (1.3 mL). The reaction was heated at reflux for 18 h. The reaction was diluted with 3:1 CHCl_3_/IPA and the layers were separated. The aqueous layer was extracted with 3:1 CHCl_3_/IPA (2x) and the combined organics were washed with water, brine, then dried (MgSO_4_), filtered, and concentrated. The crude oil was purified by using normal phase chromatography on silica gel (0–20% EtOAc/Hexanes) to provide the Boc-protected intermediate which was dissolved in DCM (9 mL) followed by addition of trifluoroacetic acid (2.1 mL, 28 mmol). After 1 h, the solvents were removed *in vacuo.* The oil was dissolved in MeOH and loaded onto SCX cartridge. The cartridge was rinsed with MeOH and 7*N* NH_3_/MeOH solution. The solvents were removed to afford the title compound (725 mg). ^1^H NMR (400 MHz, CDCl_3_) δ 6.75 (d, *J* = 8.7, 1H), 6.45 (d, *J* = 2.8, 1H), 6.41 (dd, *J* = 8.8, 2.9 Hz, 1H), 3.21–3.15 (m, 2H), 2.87–2.81 (m, 2H), 2.07–2.00 (m, 2H), 1.78–1.72 (m, 2H). LRMS: C_13_H_12_D_4_NO_3_ [M + H]^+^ calc. mass 241.2, found 241.2.

#### 6-(4-((2,3-Dihydrobenzo­[b]­[1,4]­dioxin-6-yl-2,2,3,3-d_4_)­oxy)­piperidin-1-yl-4-d)-7,8-dimethyl-[1,2,4]­triazolo­[4,3-*b*]­pyridazine **(33q, VU6025733)**

6-Chloro-7,8-dimethyl-[1,2,4]­triazolo­[4,3-*b*]­pyridazine (250 mg, 1.4 mmol), 4-deuterio-4-[(2,2,3,3-tetradeuterio-1,4-benzodioxin-6-yl)­oxy]­piperidine (345 mg, 1.4 mmol), and *N*,*N*-diisopropylethylamine (0.9 mL, 5.5 mmol) were combined in NMP (7 mL) and the vial heated at 175 °C for 18 h. The reaction was passed through a syringe filter and purified by reverse phase HLPC (20–60% MeCN/0.1% aqueous TFA) to afford the title compound (361 mg). ^1^H NMR (400 MHz, CDCl_3_) δ 8.82 (s, 1H), 6.77 (d, *J* = 8.8 Hz, 1H), 6.49 (d, *J* = 2.7 Hz, 1H), 6.45 (dd, *J* = 8.7, 2.8 Hz, 1H), 3.44–3.38 (m, 2H), 3.08–3.02 (m, 2H), 2.65 (s, 3H), 2.30 (s, 3H), 2.12–2.06 (m, 2H), 1.98–1.91 (m, 2H). ^13^C NMR (101 MHz, CDCl_3_) δ 160.1, 151.6, 145.0, 144.0, 138.5, 138.3, 133.8, 125.5, 117.6, 110.1, 105.8, 72.9–71.7 (m), 64.6–63.2 (m, 2C), 47.5 (2C), 30.6 (2C), 14.6, 13.8. HR-MS (Q-TOF, ES+) calc’d for C_20_H_18_D_5_N_5_O_3_, 387.2187; found, 387.2190.

### Molecular Pharmacology

#### Calcium Mobilization Assay

Compound-evoked increases to an EC_20_ concentration of acetylcholine (ACh) in intracellular calcium were measured using Chinese hamster ovary (CHO) cells stably expressing human, rat, dog, cyno, or minipig muscarinic receptors (M_1_–M_5_; M_2_ and M_4_ cells were cotransfected with G_qi5_). The stable cells were cultured in F12 medium containing 10% fetal bovine serum, 20 mM HEPES, 100 units/mL antibiotics/antimycotic, 0.5 mg/mL G418, and 0.2 mg/mL hygromycin (M_2_ and M_4_ G_qi5_ coexpressing cells only). All reagents used were from Life Technologies (Carlsbad, CA) unless otherwise noted.

Briefly, the day before the assay, cells (15,000 cells/20 μL/well) were plated in black-walled, clear-bottomed, 384 well plates (Greiner Bio-One, Monroe, NC) in the culture medium without G418 and hygromycin, and then incubated overnight at 37 °C in the presence of 5% CO_2_. The next day, calcium assay buffer (Hank’s balanced salt solution (HBSS), 20 mM HEPES, 2.5 mM probenecid, 4.16 mM sodium bicarbonate Sigma-Aldrich, St. Louis, MO) was prepared to dilute compounds, agonists, and Fluo-4-acetomethoxyester (Fluo-4-AM), fluorescent calcium indicator dye. Compounds were serially diluted 1:3 into 10-point concentration response curves in DMSO using the Bravo Liquid Handler (Agilent, Santa Clara, CA), transferred to a 384 well daughter plates using an Echo acoustic liquid handler (Beckman Coulter, Indianapolis, Indiana), and diluted in assay buffer to a 2X final concentration. The agonist plates were prepared using acetylcholine (ACh, Sigma-Aldrich, St. Louis, MO) concentrations for the EC_20_, EC_80_, and EC_MAX_ responses by diluting in assay buffer to a 5X final concentration. The 2X dye solution (2.3 μM) was prepared by mixing a 2.3 mM Fluo-4-AM stock in DMSO with 10% (w/v) pluronic acid F-127 in a 1:1 ratio in assay buffer. Using a microplate washer (BioTek, Winooski, VT), cells were washed with assay buffer 3 times to remove medium. After the final wash, 20 μL of assay buffer remained in the cell plates. Immediately, 20 μL of the 2X dye solution (final 1.15 μM) was added to each well of the cell plate using a Multidrop Combi dispenser (Thermo Fisher, Waltham, MA). After cells were incubated with the dye solutions for 50 min at 37 °C in the presence of 5% CO_2_, the dye solutions were removed and replaced with assay buffer using a microplate washer, leaving 20 μL of assay buffer in the cell plate, and the cell plate allowed to incubate for 10 min at 37 °C. The compound, agonist, and cell plates were placed inside the Functional Drug Screening System 7000 (FDSS7000, Hamamatsu, Japan) to measure the calcium flux. After establishment of a fluorescence baseline for 2–3 s (2–3 images at 1 Hz; excitation, 480 ± 20 nm; emission, 540 ± 30 nm), 20 μL (2X) of test compound or vehicle was added to the cells, and the response was measured. 140 s later, 10 μL (5X) of an EC_20_ concentration of ACh (Sigma-Aldrich, St. Louis, MO) or vehicle was added to the cells, and the response of the cells was measured. Approximately 125 s later, an EC_80 or_ EC_MAX_ concentration of ACh was added. Calcium fluorescence was recorded as fold over basal fluorescence and raw data were normalized to the maximal response to ACh. Compound-evoked increase in calcium response in the absence of ACh agonist was determined as ago activity of positive allosteric modulators. Compound-evoked increase in calcium response in the presence of ACh EC_20_ agonist was determined as potentiator activity of positive allosteric modulator. Potency (EC_50_) and maximum response (% ACh Max) for compounds was determined using a four-parameter logistical equation using GraphPad Prism (La Jolla, CA) or the Dotmatics software platform (Woburn, MA):

$$y=\mathrm{bottom}+\frac{\mathrm{top}-\mathrm{bottom}}{1+10^{(\mathrm{Log}\mathrm{EC}50-A)\mathrm{Hill}\mathrm{slope}}}$$

where *A* is the molar concentration of the compound; *bottom* and *top* denote the lower and upper plateaus of the concentration–response curve; HillSlope is the Hill coefficient that describes the steepness of the curve; and EC_50_ is the molar concentration of compound required to generate a response halfway between the *top* and *bottom*.

## Supplementary Material



## Notes

General methods for the synthesis and characterization of key compounds and experimental details for all assays performed (DMPK, behavioral pharmacology, ancillary pharmacology) (PDF)

## Abbreviations

ACh: acetylcholine
AD: Alzheimer’s disease
AHL: amphetamine-induced hyperlocomotion
BHB: brain homogenate binding
CL_hep_: hepatic clearance
CL_int_: intrinsic clearance
CNS: central nervous system
CYP: cytochrome P450
DMPK: drug metabolism and pharmacokinetics
FDA: food and drug administration
hM_4_: muscarinic acetylcholine receptor subtype 4, human
IND: investigational new drug
mAChR: muscarinic acetylcholine receptor
MED: minimum effective dose
NHP: nonhuman primate
PAM: positive allosteric modulator
P-gp: P-glycoprotein 1
PBL: plasma-to-brain levels
PD: pharmacodynamic
PK: pharmacokinetic
PPB: plasma protein binding
rM4: muscarinic acetylcholine receptor subtype 4, rat
SAR: structure-activity relationship
μW: microwave irradiated

## References

[1] English, B. A. ; Jones, C. K. Cholinergic Neruotransmission, In Primer on the Autonomic Nervous System, 3rd ed., Robertson, D. ; Biaggioni, I. ; Burnstock, G. , Eds.; Academic Press: London, UK, 2012, pp. 71–74. 10.1016/B978-0-12-386525-0.00014-7.

[2] Whitehouse P. J., Price D. L., Struble R. G., Clark A. W., Coyle J. T., Delong M. R. (1982). Alzheimer’s-disease and senile
dementia - Loss
of neurons in the basal forebrain. Science.

[3] Muir J. L. (1997). Acetylcholine,
aging, and Alzheimer’s disease. Pharmacol.,
Biochem. Behav.

[4] Raedler T. J., Knable M. B., Jones D. W., Urbina R. A., Gorey J. G., Lee K. S., Egan M. F., Coppola R., Weinberger D. R. (2003). In vivo
determination of muscarinic acetylcholine receptor availability in
schizophrenia. Am. J. Psychiatry.

[5] Becker R. E., Giacobini E. (1988). Mechanisms
of cholinesterase inhibition in senile dementia
of the Alzheimer type; clinical, pharmacological, and therapeutic
aspects. Drug Dev. Res.

[6] Hampel H., Mesulam M.-M., Cuello A. C., Farlow M. R., Giacobini E., Grossberg G. T., Khachaturian A. S., Vergallo A., Cavedo E., Snyder P. J. (2018). The cholinergic system in the pathophysiology
and treatment of Alzheimer’s disease. Brain.

[7] McGleenon B. M., Dynan K. B., Passmore A. P. (1999). Acetylcholinesterase
inhibitors in
Alzheimer’s disease. Br. J. Clin. Pharmacol.

[8] Balson R., Gibson P. R., Ames D., Bhathal P. S. (1995). Tacrine-induced
hepatotoxicity - tolerability and management. CNS Drugs.

[9] Langmead C. J., Watson J., Reavill C. (2008). Muscarinic acetylcholine receptors
as CNS drug targets. Pharmacol. Ther.

[10] Bonner T. I., Buckley N. J., Young A. C., Brann M. R. (1987). Identification of
a family of muscarinic acetylcholine-receptor genes. Science.

[11] Caulfield M. P. (1993). Muscarinic
receptors - characterization, coupling and function. Pharmacol. Ther.

[12] Jones C. K., Byun N., Bubser M. (2012). Muscarinic and Nicotinic
Acetylcholine
Receptor Agonists and Allosteric Modulators for the Treatment of Schizophrenia. Neuropsychopharmacology.

[13] Scarr E. (2012). Muscarinic
Receptors: Their Roles in Disorders of the Central Nervous System
and Potential as Therapeutic Targets. CNS Neurosci.
Ther.

[14] Bymaster F. P., McKinzie D. L., Felder C. C., Wess J. (2003). Use of M1-M5 muscarinic
receptor knockout mice as novel tools to delineate the physiological
roles of the muscarinic cholinergic system. Neurochem. Res.

[15] Wess J., Eglen R. M., Gautam D. (2007). Muscarinic acetylcholine receptors:
mutant mice provide new insights for drug development. Nat. Rev. Drug Discovery.

[16] Brady A. E., Jones C. K., Bridges T. M., Kennedy J. P., Thompson A. D., Heiman J. U., Breininger M. L., Gentry P. R., Yin H. Y., Jadhav S. B., Shirey J. K., Conn P. J., Lindsley C. W. (2008). Centrally
Active Allosteric Potentiators of the M4Muscarinic Acetylcholine Receptor
Reverse Amphetamine-Induced Hyperlocomotor Activity in Rats. J. Pharmacol. Exp. Ther.

[17] Pancani T., Foster D. J., Moehle M. S., Bichell T. J., Bradley E., Bridges T. M., Klar R., Poslusney M., Rook J. M., Daniels J. S., Niswender C. M., Jones C. K., Wood M. R., Bowman A. B., Lindsley C. W., Xiang Z. X., Conn P. J. (2015). Allosteric activation of M4 muscarinic
receptors improve behavioral and physiological alterations in early
symptomatic YAC128 mice. Proc. Natl. Acad. Sci.
U. S. A.

[18] Tzavara E. T., Bymaster F. P., Davis R. J., Wade M. R., Perry K. W., Wess J., McKinzie D. L., Felder C., Nomikos G. G. (2004). M-4 muscarinic
receptors regulate the dynamics of cholinergic and dopaminergic neurotransmission:
relevance to the pathophysiology and treatment of related central
nervous system pathologies. FASEB J.

[19] Nickols H. H., Conn P. J. (2014). Development of allosteric
modulators of GPCRs for treatment
of CNS disorders. Neurobiol. Dis.

[20] Bodick N. C., Offen W. W., Levey A. I., Cutler N. R., Gauthier S. G., Satlin A., Shannon H. E., Tollefson G. D., Rasmussen K., Bymaster F. P., Hurley D. J., Potter W. Z., Paul S. M. (1997). Effects of xanomeline, a selective
muscarinic receptor
agonist, on cognitive function and behavioral symptoms in Alzheimer
disease. Arch. Neurol.

[21] Shekhar A., Potter W. Z., Lightfoot J., Lienemann J., Dubé S., Mallinckrodt C., Bymaster F. P., McKinzie D. L., Felder C. C. (2018). Selective muscarinic
receptor agonist xanomeline as
a novel treatment approach for schizophrenia. Am. J. Psychiatry.

[22] FDA Approves Drug with New Mechanism of Action for Treatment of Schizophrenia. https://www.fda.gov/news-events/press-announcements/fda-approves-drug-new-mechanism-action-treatment-schizophrenia Accessed 17 March 2025.

[23] Brannan S.
K., Sawchak S., Miller A. C., Lieberman J. A., Paul S. M., Breier A. (2021). Muscarinic
Cholinergic Receptor Agonist
and Peripheral Antagonist for Schizophrenia. N. Engl. J. Med.

[24] Kenakin T., Strachan R. T. (2018). PAM-Antagonists: A Better Way to Block Pathological
Receptor Signaling?. Trends Pharmacol. Sci.

[25] Jakubik J., Bacakova L., Lisa V., ElFakahany E. E., Tucek S. (1996). Activation of muscarinic acetylcholine
receptors via their allosteric
binding sites. Proc. Natl. Acad. Sci. U. S.
A.

[26] Bubser M., Bridges T. M., Dencker D., Gould R. W., Grannan M., Noetzel M. J., Lamsal A., Niswender C. M., Daniels J. S., Poslusney M. S., Melancon B. J., Tarr J. C., Byers F. W., Wess J., Duggan M. E., Dunlop J., Wood M. W., Brandon N. J., Wood M. R., Lindsley C. W., Conn P. J., Jones C. K. (2014). Selective
activation of M_4_ muscarinic acetylcholine receptors reverses
MK-801-induced behavioral
impairments and enhances associative learning in rodents. ACS Chem. Neurosci.

[27] AbbVie Revamps Emraclidine Expectations After Mid-Stage Schizophrenia Failure. https://www.biospace.com/business/abbvie-revamps-emraclidine-expectations-after-mid-stage-schizophrenia-failure Accessed 05 January 2026.

[28] Gao, X. ; Knowles, S. L. ; Li, C. ; Man-Chu Lo, M. ; Mazzola, D. R., Jr. ; Ondeyka, D. L. 6,5-Fused heteroaryl piperidine ether allosteric modulators of the M_4_ muscarinic acetylcholine receptor. WO 2,018,112,840 A1, 2018.

[29] Besson T., Hretani M., Coudert G., Guillaumet G. (1993). Convenient
synthesis of 5-substituted-6-methoxy or 6-hydroxy-2,3-dihydro-1,4-benzodioxins
via lithiated intermediates. Tetrahedron.

[30] Temple K. J., Engers J. L., Long M. F., Watson K. J., Chang S. C., Luscombe V. B., Jenkins M. T., Rodriguez A. L., Niswender C. M., Bridges T. M., Conn P. J., Engers D. W., Lindsley C. W. (2020). Discovery of a novel 2,3-dimethylimidazo
1,2-a pyrazine-6-carboxamide
M-4 positive allosteric modulator (PAM) chemotype. Bioorg. Med. Chem. Lett.

[31] Wood M. R., Noetzel M. J., Poslusney M. S., Melancon B. J., Tarr J. C., Lamsal A., Chang S., Luscombe V. B., Weiner R. L., Cho H. P., Bubser M., Jones C. K., Niswender C. M., Wood M. W., Engers D. W., Brandon N. J., Duggan M. E., Conn P. J., Bridges T. M., Lindsley C. W. (2017). Challenges in the
development of an M4 PAM in vivo tool compound: The discovery of VU0467154
and unexpected DMPK profiles of close analogs. Bioorg. Med. Chem. Lett.

[32] See Supporting Information for full details.

