# Supplementary material for: Discovery of VU6025733 (AG06827): A Highly Selective, Orally Bioavailable, and Structurally Distinct M4 Muscarinic Acetylcholine Receptor Positive Allosteric Modulator (PAM) with Robust In Vivo Efficacy
Source: ACS Chem Neurosci. 2026 Jan 25;17(3):649–65. doi: 10.1021/acschemneuro.5c00963 (PMC12879736; doi:10.1021/acschemneuro.5c00963)
Supplement: Supplementary file 1 [file cn5c00963_si_001.pdf]

Supporting Information for

Discovery of VU6025733 (AG06827): A Highly Selective, Orally Bioavailable, and Structurally Distinct M_4 Muscarinic Acetylcholine Receptor Positive Allosteric Modulator (PAM) with Robust *In Vivo* Efficacy

Alison R. Gregro[a,b,h], Charlotte Park[a,b], Madeline F. Long[a,b,h], Logan A. Baker[a,b], Katrina A. Bollinger[a,b], Anna E. Ringuette[a,b], Li Peng[a,b], Vincent B. Luscombe[a,b], Natasha B. Billard[a,b], Alice L. Rodriguez[a,b,h], Colleen M. Niswender[a,b,e,f,h], Weimin Peng[b], Jonathan W. Dickerson[b], Jerri M. Rook[b], Jordan O'Neill[a,b], Sichen Chang[a,b,h], Harrie C.M. Boonen [g], Thomas Jensen[g], Morten S. Thomsen[g], Thomas M. Bridges[a,b], Olivier Boutaud[a,b,h], P. Jeffrey Conn[a,b,f], Darren W. Engers[a,b,h], Craig W. Lindsley[a,b,c,d,h*], Kayla J. Temple[a,b,h*]

[a]Warren Center for Neuroscience Drug Discovery, Vanderbilt University, Nashville, TN 37232, USA
[b]Department of Pharmacology, Vanderbilt University School of Medicine, Nashville, TN 37232, USA
[c]Department of Chemistry, Vanderbilt University, Nashville, TN 37232, USA
[d]Department of Biochemistry, Vanderbilt University, Nashville, TN 37232, USA
[e]Vanderbilt Kennedy Center, Vanderbilt University School of Medicine, Nashville, TN 37232, USA
[f]Vanderbilt Brain Institute, Vanderbilt University School of Medicine, Nashville, TN 37232, USA
[g]Neuroscience Drug Discovery Denmark, H. Lundbeck A/S, 9 Ottiliavej, Valby, DK-2500 Copenhagen, Denmark
[h]Vanderbilt Institute for Therapeutic Advances, Vanderbilt University, Nashville, TN 37232, USA

Table of Contents

Experimental Synthetic Procedures and Spectroscopic Data....................S2
DMPK Methods....................S7
Mini Ames Microplate Format (MPF)....................S13
In vitro Micronucleus Assay (MNvit)....................S15
Multi parametric cytotox 2 (QuadProbe assay)....................S15
hERG Patch Clamp....................S16
Bi-directional Permeability Assay in MDR1-MDCK II Cells....................S18
Rat Amphetamine-Induced Hyperlocomotion (AHL) Protocol....................S19

Experimental Synthetic Procedures and Spectroscopic Data

General Synthetic Methods.

All reactions were carried out employing standard chemical techniques. Solvents used for extraction, washing, and chromatography were HPLC grade. All reagents were purchased from commercial sources and were used without further purification.

Automated flash column chromatography was performed on a Biotage Isolera 1 or a Teledyne ISCO CombiFlash® R*f*+ system. RP-HPLC was performed on a Gilson preparative reversed-phase HPLC system comprised of a 333 aqueous pump with solvent-selection valve, 334 organic pump, GX-271 or GX-281 liquid hander, two column switching valves, and a 155 UV detector. Absorbance was typically monitored at 215 or 220 nm. Column: Phenomenex Axia-packed Gemini C18, 5 µm. Mobile phase: CH_3CN in H_2O (0.1% TFA) or CH_3CN in H_2O (0.05% *v/v* NH_4OH) under the specified gradient, then hold 95% CH_3CN in 5% aqueous phase, 50 mL/min, 23° C. All compounds were found to be >95% pure by LCMS analysis.

Safety statement*: no unexpected or unusually high safety hazards were encountered.*

General Instrumentation Methods.

All NMR spectra were recorded on a 400 MHz AMX Bruker NMR spectrometer. ^1H and ^{13}C chemical shifts are reported in δ values in ppm downfield with the deuterated solvent as the internal standard. Data are reported as follows: chemical shift, multiplicity (s = singlet, d = doublet, t = triplet, q = quartet, b = broad, m = multiplet), integration, coupling constant (Hz).

Low resolution mass spectra (LRMS) were obtained on an Agilent 6120/6150 or Waters QDa (Performance) SQ MS with ESI source. *Method A (Agilent 6120/6150):* MS parameters were as follows: fragmentor: 70, capillary voltage: 3000 V, nebulizer pressure: 30 psig, drying gas flow: 13 L/min, drying gas temperature: 350 ºC. Samples were introduced via an Agilent 1290 UHPLC comprised of a G4220A binary pump, G4226A ALS, G1316C TCC, and G4212A DAD with ULD flow cell. UV absorption was generally observed at 215 nm and 254 nm with a 4 nm bandwidth. Column: Waters Acquity BEH C18, 1.0 x 50 mm, 1.7 um. Gradient conditions: 5% to 95% CH_3CN in H_2O (0.1% TFA) over 1.4 min, hold at 95% CH_3CN for 0.1 min, 0.5 mL/min, 55 ºC. *Method B (Agilent 6120/6150):* MS parameters were as follows: fragmentor: 100, capillary voltage: 3000 V, nebulizer pressure: 40 psig, drying gas flow: 11 L/min, drying

gas temperature: 350 ºC. Samples were introduced via an Agilent 1200 HPLC comprised of a degasser, G1312A binary pump, G1367B HP-ALS, G1316A TCC, G1315D DAD, and a Varian 380 ELSD (if applicable). UV absorption was generally observed at 215 nm and 254 nm with a 4 nm bandwidth. Column: Thermo Accucore C18, 2.1 x 30 mm, 2.6 um. Gradient conditions: 7% to 95% CH_3CN in H_2O (0.1% TFA) over 1.6 min, hold at 95% CH_3CN for 0.35 min, 1.5 mL/min, 45 ºC. *Method C (Waters QDa (Performance) SQ MS)*: MS parameters were as follows: cone voltage: 15 V, capillary voltage: 0.8 kV, probe temperature: 600° C. Samples were introduced via an Acquity I-Class PLUS UPLC comprised of a BSM, FL-SM, CH-A, and PDA. UV absorption was generally observed at 215 nm and 254 nm; 4 nm bandwidth. Column: Phenomenex EVO C18, 1.0 x 50 mm, 1.7 um. Column temperature: 55° C. Flow rate: 0.4 mL/min. Default gradient: 5% to 95% CH_3CN (0.05% TFA) in H_2O (0.05% TFA) over 1.4 min (curve 6), hold at 95% CH_3CN for 0.1 min. "Polar" (2% to 70% CH_3CN (0.05% TFA) in H_2O (0.05% TFA) over 0.8 min (curve 6), transition to 95% CH_3CN over 0.1 min (curve 6), hold at 95% CH_3CN for 0.6 min.) and "Non-Polar" (40% to 95% CH_3CN (0.05% TFA) in H_2O (0.05% TFA) over 1.4 min (curve 6), hold at 95% CH_3CN for 0.1 min.) gradients were also available. *Method D (Waters QDa (Performance) SQ MS):* MS parameters were as follows: cone voltage: 15 V, capillary voltage: 0.8 kV, probe temperature: 600° C. Samples were introduced via an Acquity I-Class PLUS UPLC comprised of a BSM, FL-SM, CH-A, and PDA. UV absorption was generally observed at 215 nm and 254 nm with a 4 nm bandwidth. Column: Phenomenex EVO C18, 1.0 x 50 mm, 1.7 um. Column temperature: 55° C. Flow rate: 0.4 mL/min. Default gradient: 5% to 95% CH_3CN in H_2O (5 mM NH_4HCO_3) over 1.4 min (curve 6), hold at 95% CH_3CN for 0.1 min. "Polar" (2% to 70% CH_3CN in H_2O (5 mM NH_4HCO_3) over 0.8 min (curve 6), transition to 95% CH_3CN over 0.1 min (curve 6), hold at 95% CH_3CN for 0.6 min.) and "Non-Polar" (40% to 95% CH_3CN in H_2O (5 mM NH_4HCO_3) over 1.4 min (curve 6), hold at 95% CH_3CN for 0.1 min.) gradients were also available.

High resolution mass spectra (HRMS) were obtained on an Agilent 6540 UHD Q-TOF with ESI source. MS parameters were as follows: fragmentor: 150, capillary voltage: 3500 V, nebulizer pressure: 60 psig, drying gas flow: 13 L/min, drying gas temperature: 275 ºC. Samples were introduced via an Agilent 1200 UHPLC comprised of a G4220A binary pump, G4226A 3 ALS, G1316C TCC, and G4212A DAD with ULD flow cell. UV absorption was observed at 215 nm and 254 nm with a 4 nm bandwidth. Column: Agilent Zorbax Extend C18, 1.8 µm, 2.1 x 50 mm. Gradient conditions: 5% to 95% CH_3CN in H_2O (0.1% formic acid) over 1 min, hold at 95% CH_3CN for 0.1 min, 0.5 mL/min, 40 ºC.

General Procedure for the Preparation of Analog **33q**.

*Synthesis of 6-chloro-7,8-dimethyl-[1,2,4]triazolo[4,3-*b*]pyridazine (Intermediate **32, Scheme 5**):*

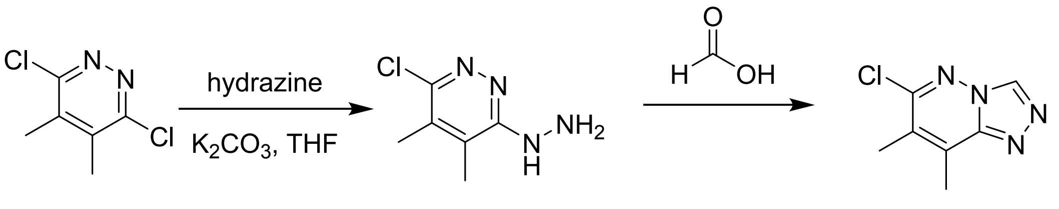

Step 1 (Intermediate S1): 3,6-Dichloro-4,5-dimethylpyridazine (1.0 g, 5.6 mmol) and potassium carbonate (79 mg, 0.56 mmol) were dissolved in THF (28 mL) before the addition of hydrazine (890 µL, 28.2 mmol) dropwise under N_2 atmosphere. The reaction mixture was heated to reflux. After 72 hours, the reaction was concentrated *in vacuo* and used without further purification (975 mg). LRMS: $C_6H_9ClN_4$ $[M+H]^+$ calc. mass 173.0, found 173.3.

Step 2 (Intermediate 32): The crude residue of 3-chloro-6-hydrazineylidene-4,5-dimethyl-1,6-dihydropyridazine (975 mg, 5.6 mmol) and formic acid (1.06 mL) were added to a sealed vessel. After heating at 100 °C for one hour, the mixture was concentrated *in vacuo*. The crude material was dissolved in DCM, washed with 10 % aqueous K_2CO_3 and back extracted with DCM (2×). The combined organic layers were dried ($MgSO_4$), filtered, and concentrated in vacuo. The crude material was purified using flash chromatography on silica gel to afford the title compound (755 mg). ^{1}H NMR (400 MHz, $CDCl_3$) δ 8.89 (s, 1H), 2.67 (s, 3H), 2.36 (s, 3H). LRMS: $C_7H_7ClN_4$ $[M+H]^+$ calc. mass 183.0, found 183.4.

Synthesis of tert-*butyl 4-(tosyloxy)piperidine-1-carboxylate-4-*d *(Intermediate **35, Scheme 5**):*

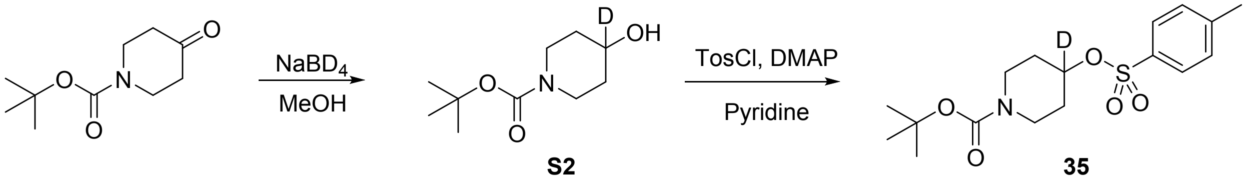

Step 1 (Intermediate S2): To a 0 °C solution of 1-*tert*-butyl-4-piperidone (10 g, 50 mmol) in methanol (250 mL) was added sodium borodeuteride (3.2mL, 100 mmol). The resulting mixture was stirred for 4 h at room temperature. The reaction was quenched with saturated NH_4Cl and extracted with EtOAc (3x). The combined organic layers were dried ($MgSO_4$), filtered, and concentrated to afford the title compound (10 g). ^{1}H NMR (400 MHz, $CDCl_3$) δ 3.85 (d, J = 12.4 Hz, 2H), 3.06-3.00 (m, 2H), 1.87-1.82 (m, 2H), 1.48-1.42 (m, 2H), 1.46 (s, 9H). LRMS: $C_{10}H_{18}DNO_3$ $[M+H]^+$ calc. mass 203.1, found 147.3 (loss of *t*Bu observed).

Step 2 (Intermediate 35): To a suspension of *tert*-butyl 4-hydroxypiperidine-1-carboxylate-4-*d* (10 g, 49 mmol) and 4-dimethyl-aminopyridine (0.6 g, 4.9 mmol) in pyridine (45 mL) was added tosyl chloride (11.8 g, 62 mmol). The mixture stirred at room temperature for 18 h. The reaction was quenched with saturated aqueous $NaHCO_3$ solution and extracted with EtOAc (2x). The combined organic layers were washed with water (2x), brine (2x), dried ($MgSO_4$), filtered, and concentrated. The crude oil was purified via normal-phase chromatography on silica gel (0-20% EtOAc/Hexanes) to provide the title compound (14.3 g). ^1H NMR (400 MHz, $CDCl_3$) δ 7.79 (d, J = 8.4 Hz, 2H), 7.34 (d, J = 8.0 Hz, 2H), 3.61-3.55 (m, 2H), 3.28-3.22 (m, 2H), 2.45 (s, 3H), 1.79-1.73 (m, 2H), 1.70-1.64 (m, 2H), 1.43 (s, 9H). LRMS: $C_{17}H_{24}DNO_5S$ $[M+Na]^+$ calc. mass 379.1, found 379.4.

*Synthesis of 2,3-dihydrobenzo[*b*][1,4]dioxin-2,2,3,3-*d_4*-6-ol (Intermediate **8**, **Scheme 2**):*

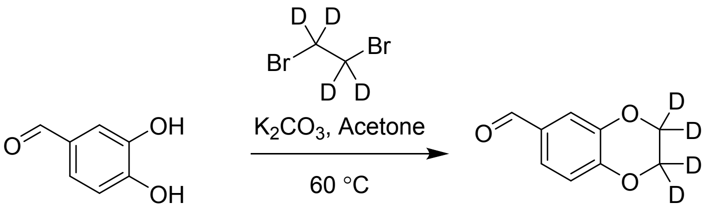

7 **S3**

Intermediate S3: In a 500 mL round bottom flask were combined 3,4-dihydroxybenzaldehyde (21.3 g), 1,2-dibromoethane-1,1,2,2-d_4 (14.1 mL), and potassium carbonate (65.0 g) in acetone (515 mL). The reaction was heated to reflux for 18 h. The reaction was diluted with EtOAc, filtered through Celite®, and the filtrate was concentrated *in vacuo*. The crude sample was purified by flash chromatography on silica gel (0-40% EtOAc/Hexanes) to afford the title compound 15.4 g. ^1H NMR (400 MHz, $CDCl_3$) δ 9.82 (s, 1H), 7.41-7.38 (m, 2H), 6.97 (d, J = 8.8 Hz, 1H). LRMS: $C_9H_4D_4O_3$ $[M+H]^+$ calc. mass 169.1, found 169.2.

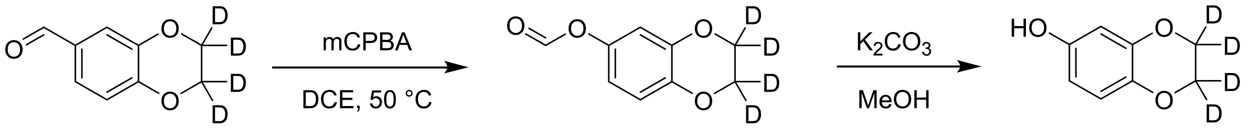

S3 **S4** **8**

Step 1 (Intermediate S4): To a solution of 2,3-dihydrobenzo[*b*][1,4]dioxine-2,2,3,3-*d_4*-6-carbaldehyde (15.4 g) in DCE (250 mL) was added 3-chloroperoxybenzoic acid (47.6 g, <77% CAS#937-14-4; Sigma-Aldrich). The reaction mixture was heated to 50 °C for 18 h. The reaction was diluted with DCM and saturated $NaHCO_3$ solution. After the layers were separated, the aqueous layer was re-extracted with DCM

(2×), and the combined organic layers were washed with brine, dried ($MgSO_4$), filtered, and concentrated to afford 2,3-dihydrobenzo[*b*][1,4]dioxin-6-yl-2,2,3,3-*d*$_4$ formate (15.1 g). LRMS: $C_9H_4D_4O_4$ $[M+H]^+$ calc. mass 185.1, found 185.2.

Step 2 (Intermediate 8): 2,3-Dihydrobenzo[*b*][1,4]dioxin-6-yl-2,2,3,3-*d*$_4$ formate (15.1 g) was dissolved in methanol (250 mL) and potassium carbonate (15.5 g) was added. After 3 h, the solvent was removed and water/DCM (1:1) were added. The aqueous layer was slowly acidified by dropwise addition of 6 *N* aqueous HCl to pH < 4. The layers were separated, the aqueous layer was extracted with DCM (2×) and the combined organic layers were washed with brine, dried ($MgSO_4$), filtered, and concentrated. The filtrate was purified by flash chromatography on silica gel (0-40% EtOAc/Hexanes). The desired fractions were concentrated and azeotroped (2× with toluene) to afford the title compound (12.3 g). ^{1}H NMR (400 MHz, $CDCl_3$) δ 6.72 (d, J = 8.8 Hz, 1H), 6.39 (d, J = 2.9 Hz, 1H), 6.34-6.31 (dd, J = 8.7, 2.9 Hz, 1H), 4.64 (s, 1H). LRMS: $C_8H_4D_4O_3$ $[M+H]^+$ calc. mass 157.1, found 157.2.

*Synthesis of 4-((2,3-Dihydrobenzo[*b*][1,4]dioxin-6-yl-2,2,3,3-*d*$_4$)oxy)piperidine-4-*d* (Intermediate **36** (**Scheme 5**):*

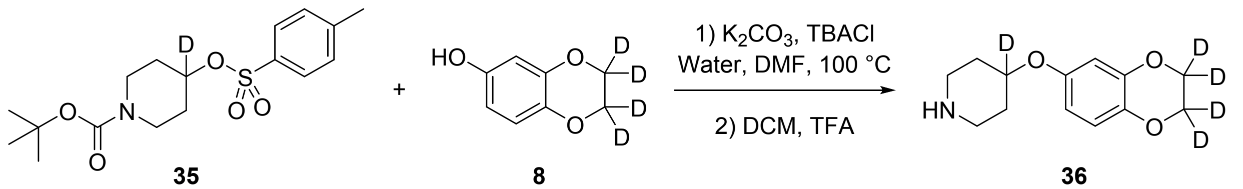

To a round bottom flask were added 2,2,3,3-tetradeuterio-1,4-benzodioxin-6-ol (1.0 g, 6.7 mmol), *tert*-butyl 4-deuterio-4-(p-tolylsulfonyloxy)piperidine-1-carboxylate (2.0 g, 5.6 mmol), potassium carbonate (2.4 g, 16.8 mmol), and tetrabutylammonium chloride (0.31 g, 1.1 mmol) in water (25 mL) and DMF (1.3 mL). The reaction was heated at reflux for 18 h. The reaction was diluted with 3:1 $CHCl_3$/IPA and the layers were separated. The aqueous layer was extracted with 3:1 $CHCl_3$/IPA (2x) and the combined organics were washed with water, brine, then dried ($MgSO_4$), filtered, and concentrated. The crude oil was purified by using normal phase chromatography on silica gel (0-20% EtOAc/Hexanes) to provide the Boc-protected intermediate which was dissolved in DCM (9 mL) followed by addition of trifluoroacetic acid (2.1 mL, 28 mmol). After 1 h, the solvents were removed *in vacuo.* The oil was dissolved in MeOH and loaded onto SCX cartridge. The cartridge was rinsed with MeOH and 7*N* NH_3/MeOH solution. The solvents were removed to afford the title compound (725 mg). ^{1}H NMR (400 MHz, $CDCl_3$) δ 6.75 (d, J = 8.7, 1H), 6.45 (d, J = 2.8, 1H), 6.41 (dd, J = 8.8, 2.9 Hz, 1H), 3.21-3.15 (m, 2H), 2.87-2.81 (m, 2H), 2.07-2.00 (m, 2H), 1.78-1.72 (m, 2H). LRMS: $C_{13}H_{12}D_4NO_3$ $[M+H]^+$ calc. mass 241.2, found 241.2.

*Preparation of 6-(4-((2,3-dihydrobenzo[*b*][1,4]dioxin-6-yl-2,2,3,3-*d_4*)oxy)piperidin-1-yl-4-d)-7,8-dimethyl-[1,2,4]triazolo[4,3-*b*]pyridazine (**33q, VU6025733, Scheme 5**):*

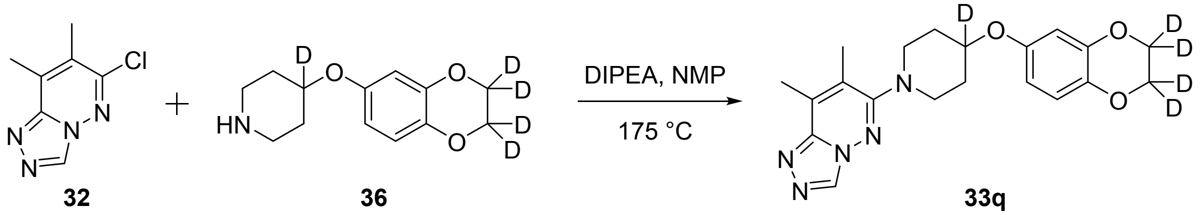

6-Chloro-7,8-dimethyl-[1,2,4]triazolo[4,3-*b*]pyridazine (250 mg, 1.4 mmol), 4-deuterio-4-[(2,2,3,3-tetradeuterio-1,4-benzodioxin-6-yl)oxy]piperidine (345 mg, 1.4 mmol), and *N*,*N*-diisopropylethylamine (0.9 mL, 5.5 mmol) were combined in NMP (7 mL) and the vial heated at 175 °C for 18 h. The reaction was passed through a syringe filter and purified by reverse phase HLPC (20-60% MeCN/0.1% aqueous TFA) to afford the title compound (361 mg). ^{1}H NMR (400 MHz, $CDCl_3$) δ 8.82 (s, 1H), 6.77 (d, J = 8.8 Hz, 1H), 6.49 (d, J = 2.7 Hz, 1H), 6.45 (dd, J = 8.7, 2.8 Hz, 1H), 3.44-3.38 (m, 2H), 3.08-3.02 (m, 2H), 2.65 (s, 3H), 2.30 (s, 3H), 2.12-2.06 (m, 2H), 1.98-1.91 (m, 2H). ^{13}C NMR (101 MHz, CDCl3) δ 160.1, 151.6, 145.0, 144.0, 138.5, 138.3, 133.8, 125.5, 117.6, 110.1, 105.8, 72.9 – 71.7 (m), 64.6 – 63.2 (m, 2C), 47.5 (2C), 30.6 (2C), 14.6, 13.8. HR-MS (Q-TOF, ES+) calc'd for $C_{20}H_{18}D_5N_5O_3$, 387.2187; found, 387.2190.

<u>DMPK Methods</u>

Animal care and use: All animal study procedures were approved by the Institutional Animal Care and Use Committee and were conducted in accordance with the National Institutes of Health regulations of animal care covered in Principles of Laboratory Animal Care (National Institutes of Health).

In-life phase

<u>Intravenous pharmacokinetics and plasma-brain level determination (IV PK PBL) **VU6025733**</u>

Compounds were formulated as a solution in ethanol, PEG400, and saline (1:4:5 v/v, respectively) at a concentration of 1 mg/mL and administered as a single 0.2 mg/kg IV dose (1 mL/kg) to male, Sprague Dawley rats (n = 1; 342 gram body weights) via injection into a surgically-implanted jugular vein catheter. For dog PK, **VU6025733** was formulated in 10% (2-Hydroxypropyl)-beta-cyclodextrin (HPbCD) at a concentration of 0.5 mg/ml and administered as a single dose of 0.5 mg/kg to male beagles (1 ml/kg, n=3). Blood samples were collected serially from a surgically implanted carotid artery catheter in each animal

over multiple post-administration time points (0.033, 0.117, 0.25, 0.5, 1, 2, 4, 7, and 24 hours) into chilled, K2EDTA anticoagulant-fortified tubes and immediately placed on wet ice. The blood samples were then centrifuged (1700 rcf, 5 minutes, 4 °C) in order to obtain plasma samples, which were stored at -80 °C until analysis by LC-MS/MS. A control compound was co-injected with every cassette to assess validity of the PK data. If PK parameters for the control fall outside our historical range, the cassette was repeated. Historical ranges for the control are as follows: $t_{1/2}$ between 2-5 hr, MRT between 2-5 hr, CL_p between 5 and 15, mL/min/kg V_{ss} between 0.5 and 3 L/kg, and K_p between 0.7 and 1.5.

For determination of the brain over plasma ratio (K_p), compounds were formulated in 8% ethanol, 32% PEG400 and 60% DMSO (v/v/v) and administered as a single 0.2 mg/kg IV dose (1 mL/kg) to male, Sprague Dawley rats (n = 1; 316 gram body weights) via injection into a surgically-implanted jugular vein catheter. At 15 min post dosing, blood sample was collected serially (i.e., terminally) into chilled, K2EDTA anticoagulant-fortified tube and immediately placed on wet ice. The blood sample was then centrifuged (1700 rcf, 5 minutes, 4 °C) to obtain plasma sample. At the same post-administration time point, whole brain sample was obtained by rapid dissection, rinsed with PBS, and immediately frozen in individual tissue collection box (dry ice). All brain and plasma samples were stored at -80 °C until analysis by LC-MS/MS.

For PO PK studies, **VU6025733** was formulated in 10% Tween 80 in water and administered orally as a single dose of 10 mg/kg to male Sprague Dawley rats (10 ml/kg, n=2) that were fasted overnight. Blood samples were collected serially from a surgically implanted carotid artery catheter in each animal over multiple post-administration time points (0.25, 0.5, 1, 2, 4, 7, and 24 hours) into chilled, K2EDTA anticoagulant-fortified tubes and immediately placed on wet ice. The blood samples were then centrifuged (1700 rcf, 5 minutes, 4 °C) in order to obtain plasma samples, which were stored at -80 °C until analysis by LC-MS/MS.

Samples preparation for bioanalysis

Plasma samples from the in-life phase of the study were thawed at ambient temperature (benchtop), and then aliquots (20 µL per sample) were transferred to a 96-shallow-well (V-bottom) plate. Matrix-matched quality control (QC) samples and a standard curve of **VU6025733** (1 mg/mL DMSO stock solution) were prepared in blank rat plasma (K2EDTA-treated) or blank brain homogenate via serial dilution and transferred (20 µL each) to the plate along with multiple blank plasma and brain homogenate samples. Acetonitrile (120 µL) containing IS (10 nM carbamazepine) was added to each well of the plate to precipitate protein. The plate was then centrifuged (4000 rcf, 5 minutes, ambient temperature), and resulting supernatants (60 µL each) were transferred to a new 96-shallow-well (V-bottom) plate containing an equal volume (60 µL per well) of water (Milli-Q purified). The plate was then sealed in preparation for LC-MS/MS analysis.

Preparation of brain samples was identical to that of plasma samples except for the following modifications. While thawing, brains were weighed (inside their collection boxes using a universal empty collection box tare weight) and then subjected to mechanical homogenization (Mini-BeadBeater™, BioSpec Products, Inc., Bartlesville, OK) in the presence of zirconia/silica beads (1.0 mm) and extraction buffer (isopropanol:water, 7:3, v/v; 3 mL per sample, corrected for post-quantitation). Homogenized brain samples were then centrifuged (4000 rcf, 5 minutes, ambient temperature), and 5 µL of the supernatant was diluted in 15 µL of blank plasma for quantification of the analyte. The plasma standard curve and QCs were used for compounds quantitation in brain.

Binding in plasma from rat and human.

Determination of compounds' fraction unbound (f_u) in plasma from rat and human was conducted *in vitro* via equilibrium dialysis using HTDialysis membrane plates. Dialysis membranes (four paired strips per HTD assay) were hydrated as described by the manufacturer and inserted into the HTD plate, which was assembled and prepared for sample addition by the dispensing of blank buffer (DPBS, 100 µL/well) into the 'top half' of each membrane-split well. Each compound was diluted into plasma from each species (5 µM final concentration), which was aliquoted in triplicate to the 'bottom half' of the prepared HTD plate wells. The HTD plate was sealed and incubated for 6 hours at 37 °C. Following incubation, each well (both top and bottom halves) were transferred (20 µL) to the corresponding wells of a 96-shallow-well (V-bottom) plate. The daughter plates were then matrix-matched (buffer side wells received equal volume of plasma, and plasma side wells received equal volume of buffer), and extraction solution (120 µL; acetonitrile containing 50 nM carbamazepine as IS) was added to all wells of both daughter plates to precipitate protein and extract test article. The plates were then sealed and centrifuged (3500 rcf) for 10 minutes at ambient temperature. Supernatant (60 µL) from each well of the daughter plates was then transferred to the corresponding wells of new daughter plates (96-shallow-well, V bottom) containing water (Milli-Q, 60 µL/well), and the plates were sealed in preparation for LC-MS/MS analysis (see below).

f_u was calculated as (analyte to IS MS peak area ratio from Trans-buffer side) / (analyte to IS MS peak area ratio from Cis-plasma side). Mean values for each species were calculated from 3 replicates. A positive control (warfarin) was included in every analytical batch to assess assay reproducibility. **Tables S1 and S2** show the historical range of unbound fraction in plasma and brain homogenate for each species tested. If f_u values for the control were outside these ranges, the assay was repeated.

Table S1. Warfarin control data for plasma protein binding assay.

	**Human f_u**	**Rat f_u**	**Mouse f_u**
**Average:**	0.073	0.030	0.222
**Maximum:**	0.111	0.057	0.299
**Minimum:**	0.048	0.011	0.173
**Range:**	0.062	0.045	0.126

Binding in brain homogenate from rat.

Determination of fraction unbound (f_u) in brain homogenate from rat was conducted using the same methodology and procedure than described for plasma protein binding assay with the following modifications: 1) a final compound concentration of 1 µM was used, 2) naïve rat brains were homogenized in DPBS (1:3 composition of brain: DPBS, w/w) using a Mini-Bead Beater™ machine in order to obtain brain homogenate.

The diluted fraction unbound (f_{u2}) in brain was calculated as (analyte to IS MS peak area ratio from Trans-buffer side) / (analyte to IS MS peak area ratio from Cis-brain homogenate side). Undiluted fraction unbound for the brain was calculated using the following equation:

$$f_u = \frac{1/4}{\left\{\left(\frac{1}{f_{u2}}\right) - 1\right\} + 1/4}$$

Mean values for each species were calculated from 3 replicates.

Table S2. Warfarin control data for brain homogenate binding assay.

	**Rat f_u**
**Average:**	0.266
**Maximum:**	0.351
**Minimum:**	0.175
**Range:**	0.175

Intrinsic Clearance in Rat and Human Liver Microsomes

The *in vitro* intrinsic clearance (CL_{int}) was investigated in commercially obtained hepatic microsomes from rat and human donors using the substrate depletion (i.e., loss-of-parent vs. time, or $t_{1/2}$ method) approach with analyte detection via liquid chromatography-tandem mass spectrometry (LC-

MS/MS). For each species, mean %parent remaining values at each time point were calculated from replicates raw data (analyte:IS peak area ratios) and used to determine *in vitro* $t_{1/2}$ and CL_{int}.

Experiments were carried out using a robot-assisted (TECAN model Evo 200). Compound was incubated (1 μM final concentration) in buffer (100 mM potassium phosphate pH 7.4 with 3 mM $MgCl_2$) containing hepatic microsomes (0.5 mg/mL final concentration) from multiple species, discretely, at 37 °C under constant orbital shaking. After 5 minutes (pre-incubation), reactions were initiated by addition of nicotinamide adenine dinucleotide phosphate (NADPH, 1 mM final concentration). At selected time intervals (0, 3, 7, 15, 25, and 45 minutes) post-addition of NADPH, aliquots (50 μL) were taken and placed into a 96-shallow-well plate containing ice cold acetonitrile (150 μL) with carbamazepine (IS, 50 nM). The plates were then centrifuged (3000 rcf at 4 °C) for 10 minutes. The supernatants were transferred to a new 96-shallow-well daughter plate and diluted (1:1 v/v) with water (Milli-Q filtered). The plates were then sealed in preparation for LC-MS/MS analysis (see below).

Raw LC-MS/MS peak area data generated from the assay samples were used to construct natural log-transformed %parent remaining vs. time plots (using t = 0 minute post-NADPH addition sample data as starting point set to 100%). *In vitro* compound half-life ($t_{1/2}$) values were obtained using the following equation:

$$t_{1/2} = \frac{Ln(2)}{k}$$

Where k is the slope from linear regression analysis of the natural log-transformed data (using means from all replicates at each time point). Resulting $t_{1/2}$ values were then used to calculate hepatic CL_{int} values according to the following equation and with the use of species-specific scale-up factors for liver weight (grams) per total body weight (kg):

$$CL_{int} = \frac{0.693}{in\ vitro\ t_{1/2}}\ x\ \frac{1\ mL\ incubation}{0.5\ mg\ microsomes}\ x\ \frac{45\ mg\ microsomes}{1\ gram\ liver}\ x\ \frac{{}^{a}\ gram\ liver}{kg\ body\ wt}$$

[a]Scale-up factors used are 45 (rat) and 20 (human).[2]

Predicted hepatic clearance (CL_{hep}) was calculated using the following equation:

$$CL_{hep} = \frac{Q_h * CL_{int}}{Q_h + CL_{int}}$$

Q_h represents hepatic blood flow (mL/min/kg): 21 for human, 70 for rat, and 90 for mouse.

A positive control (verapamil) was included in every analytical batch to assess assay reproducibility. **Table S3** shows the historical range of Intrinsic clearance for each species tested. If CL_{int} values for the control were outside these range, the assay was repeated.

Table S3. Verapamil control data for intrinsic clearance assay

	**Human CL_{hep}**	**Rat CL_{hep}**	**Mouse CL_{hep}**
**Average:**	19.0	64.1	84.6
**Maximum:**	19.8	65.9	87.1
**Minimum:**	17.1	57.7	79.9
**Range:**	2.63	8.20	7.15

LC-MS/MS Analysis

Prepared samples were injected (10 µL each) onto an AB Sciex Triple Quad 4500 mass spectrometer system with an Agilent 1260 Infinity II pump and autosampler. Mass spectrometer conditions are described in **Table S4.** Quantitation of compounds was performed via AB Sciex Multiquant software using the raw analyte:IS peak area ratios. The typical detection range was 0.5 ng/mL to ≥ 5,000 ng/mL utilizing a quadratic equation regression with 1/x2 weighting.

Correction for dilution of all brain samples (in extraction buffer and subsequently in blank plasma, as previously described) was performed post-quantitation. The corrections for dilution in extraction buffer employed correction factors specific to each brain weight (not shown).

Table S4. **LC-MS/MS Conditions**

Injection volume	10 µL	
Mobile phase A	0.5% Formic Acid in Water	
Mobile phase B	0.5% Formic Acid in Acetonitrile	
Flowrate	0.5 mL/min	
Gradient	Time	% Mobile Phase B
	0.0	5
	0.2	5
	0.8	95
	1.5	95
	1.7	5
	2.7	Stop
Column	Fortis C18 (50 x 3.0 mm, 3 µm)	
Data collection and analysis software/version	Analyst v. 1.7.1	
Ionization mode	Positive Electrospray	
Curtain gas (psi)	40	
GS1 (psi)	40	
GS2 (psi)	40	
Capillary voltage (V)	5500	
Source TurboIonSpray® temp. (°C)	500	

<u>Mini Ames Microplate Format (MPF)</u>

The bacterial mutation test (BMT) used by Gentronix is the Ames MPF™ assay, a modified version of the well-known Ames test, that uses liquid bacterial culture rather than agar plates. THE MPF assay is based on the fluctuation method cited in OECD Guideline 471 (OECD Guideline for testing of Chemicals, Bacterial Reverse Mutation Test), but incubation and selection of revertant colonies is performed in 384-well microplates using a colorimetric endpoint. A major advantage of this method over the ‘traditional’ Ames test technique is the significant reduction in the quantity of test compound requires.

Basic Protocol: In 24-well microplates, histidine auxotrophic bacteria are exposed to 6 doses of a test compound in the absence of presence of S9, as well as both a positive and negative control. Exposures are performed in triplicate (1 × 24-well plate for 1 compound with 1 S9 condition) and last for 90 minutes with shaking at 37 °C. The exposure medium contains sufficient histidine to support ~2 cell divisions during the exposure period. After the 90 minutes incubation, exposure cultures are diluted in histidine-free medium containing a pH indicator dye. The diluted culture from each treatment (1 well of a 24-well plate) is then aliquoted into 48 wells (an eighth sector) of a 384-well microplate, such that a single 384-well microplate represents a single replicate of a compound test (either with or without S9 metabolic activation), and hence the contents of 1 complete 24-well plate are transferred to a 3 × 384-well microplates.

After a further incubation of 48 hours at 37 °C, 384-well plates are scored by counting colored wells (see **Figure S1**). Cells that have mutated back to histidine prototrophy, either spontaneously or as a result of test compound / positive control treatment, are able to grow into colonies. In wells where this occurs, the cells’ metabolism reduces the pH of the medium changing the indicator color from purple to yellow. These yellow revertant wells are counted for each dose and compared to the zero-dose (vehicle-treated control).

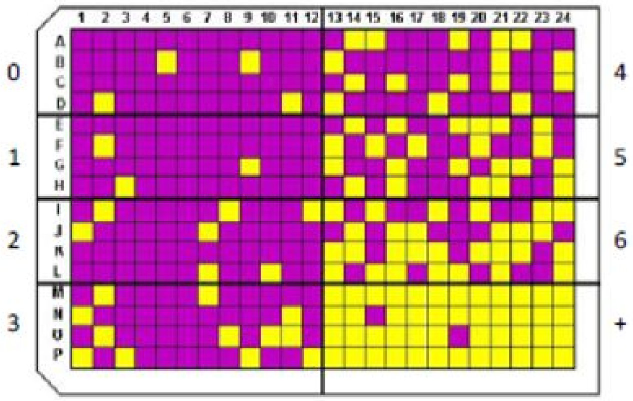

Figure S1. Representation of a revertant colony selection plate showing 48-well sectors for each dose (labelled 0 – 6) and the positive control (+). Purple color signifies no reversion in a well whilst yellow indicates revertants present.

S9 Treatment: Test compound exposures in the presence of S9 metabolic activation use Aroclor-induced rat liver S9 at a final concentration in the exposure culture of 4.5%. Prior to inclusion in the assay, stock S9 is used to prepare a 30% S9 mix in a buffered co-factor solution. This S9 mix is incorporated into the exposure medium / culture immediately prior to its combination with the test compound doses and controls in the 24-well exposure microplate.

Strains: The bacterial strains employed in this study are shown in **Table S5**

Table S5. The Salmonella typhimurium strains used in this study with relevant genotype information. (Key: rfa = mutation leading to defective cell coating and hence grater permeability; uvrB = mutation in accurate excision repair system leading to more error-prone DNA repair; pKM101 = R-factor plasmid enhances error-prone recombinational DMA repair of induced mutagenesis and confers ampicillin resistance on the host cell.)

Strain	Mutation	Type	Target	Cell Wall	Repair	pKM101
**TA98**	hisD3052	Frameshifts	GCGCGCGC	rfa	uvrB	yes
**TA100**	hisG46	Base-pair substitution	GGG	rfa	uvrB	Yes

Positive Controls: The positive controls and final assay concentrations are provided for each strain ±S9 in **Table S6**.

Table S6. Positive control compounds and test concentrations used with each strain.

<table>
<tr><th rowspan="2">Stain</th><th colspan="2">Without S9</th><th colspan="2">With S9</th></tr>
<tr><th>Compound</th><th>Concentration (µg/µL)</th><th>Compound</th><th>Concentration (µg/µL)</th></tr>
<tr><td>TA98</td><td>2-Nitrofluorene</td><td>2</td><td rowspan="2">2-Aminoanthracene</td><td rowspan="2">5.0</td></tr>
<tr><td>TA100</td><td>4-Nitroquinoline-N-oxide</td><td>0.1</td></tr>
</table>

Calculations: Positive wells (yellow color change) are scored for each 384-well revertant selection plate and used to calculate the 'mean number of positive wells per compound dose'- the average number of yellow wells from 3 replicates of a dose.

The 'standard deviation of positive wells per dose' is calculated as the standard deviation of each 'mean number of positive wells'.

The background level of reversion is calculated as the mean number of positive wells from the vehicle-treated controls (dose 0 in Figure S1) from triplicate 384-well plates. The standard deviation for this mean is calculated and added to the mean to generate the 'baseline' for background reversion. A baseline that is calculated to be ,1.0 is automatically reset to 1.0.

The ratio of the mean number of positive wells for a compound dose to the baseline yields the 'fold induction over the baseline'.

Data Interpretation: Certain data acceptance criteria must be met for an assay to be considered valid, as follows:

- The background level of reversion observed in the negative controls must fall within the expected range defined by the Ames MPF assay's originating laboratory.
- The background level of reversion observed in the negative controls must fall withing the historical control range for this laboratory.
- Positive controls are expected to yield ≥ 25 positive wells per treatment.

A compound is classed as a bacterial mutagen in the assay if:

- It produces a clear dose-response in the mean positive wells per dose
- P values from a 1-sided, unpaired Student's t-test are also used to evaluate results

And/or

- It yields multiple fold inductions of >2.0 over the baseline.

Conversely, fold inductions of revertants over the baseline but below 2.0-fold are not considered indicative of mutagenicity.

In vitro **Micronucleus Assay (MNvit)**

Purpose: To test if a compound has genotoxic potential by assessing if the compound causes DNA damage in the form of micronuclei, either by a direct (clastogenic) or indirect (aneugenic) interaction both with and without metabolic activation using rat liver microsomal fraction S9.

Assay details: TK6 cells are dosed with 10 concentrations of the compound in doublets in 1%DMSO with and without metabolic activation (S9) in 96-well plates. Following criteria (according to the ICH S2 guideline) are considered when choosing the test concentrations: A relative survival of 0.45-0.55 (Relative Nuclei Count) must be reached, if not possible the compound must be tested up to 1 mM or until the compound is no longer soluble under assay conditions. The cells are incubated with the compound at 37^{0}C, 5% CO_2 for 24 hours, when S9 is not included and for 3 hours with S9 followed by 21 hours without compound and S9.

Using the MicroFlow, in vitro kit from Litron Laboratories, the cells undergo a dual staining procedure, where the cells first are stained with EMA (ethidium monoazide), a DNA stain which only stains the DNA from cells with compromised cell membranes (stains the DNA from late apoptotic, necrotic and dead cells) and secondly in a lysing solution, SYTOX Green which stains all DNA from the lysed cells.

25 µL of the stained, lysed cells are analyzed on a flow cytometer and after applying gating procedures, the final parameters used to assess the induction of micronuclei is % micronuclei (%MN), % EMA positives (%EMA) and nuclei count.

On each plate, 8 negative control samples (1% DMSO) and 8 positive control samples (MMS for MN-S9 and CPA for MN+S9) are included. The fold increase in MN and EMA and RNC are calculated relative to the average of the negative controls. Cytotox LEC is assessed from either the concentration where fold EMA is larger than or equal to 3.5 and/or RNC is between 0.45 and 0.55. Genotox LEC is the concentration where fold MN is larger than or equal to 4.5. If the Genotox LEC is lower than the Cytotox LEC the outcome is reported at negative, if the Genotox LEC is equal to or larger than the Cytotox LEC the outcome is reported positive.

Multi parametric cytotox 2 (QuadProbe assay)[3]

Purpose: The primary assay for cytotoxicity is the multi-parametric Cytotox (Quadprobe) assay. The assay is performed in human HepG2 cells (human liver cancer cell line) to model potential liver toxicity of compounds in humans. Liver toxicity is one of the major causes for attrition of drugs in clinical testing and use.

By using four different fluorescent probes for assessing various aspects of cell health, the cytotoxicity of a compound is estimated. The analysis is performed using an Operetta (Perkin Elmer) High Content Screen (HCS) instrument. The cell features are: Cell survival (nuclear count), DNA staining (nuclear area), lysosomal activity, mitochondrial function (mitochondrial membrane potential), increase or degradation of mitochondria (mitochondrial mass (mitochondrial area)) and leakage across the plasma membrane due to beginning cell death (membrane integrity).

Table S7. Cytotox assay set-up.

Plates	96 wells Optical Btm Plt Polymerbase, 165305
Plates coated with	Poly-L-Lysine No.: P8920-500ml, Sigma Aldrich
Tested concentrations	100, 67, 44, 30, 20, 13, 9, 1, 0.1 and 0.01 µM*
Incubation time	24 hours in 37°C, 5% CO_2
Fluorescent probes	Hoechst 33342 (1.62 µM), Lyso Tracker Green (50 nM), Mito Tracker Orange (50 nM) and TOTO-3 (1 µM)**
Blanks	DMSO
Control compounds	Disulfiram and Chlorpromazine
Fixated in	4% Paraformaldehyde
Data read on	ArrayScan Vti (HCS), 20x

*Compounds are tested up to a concentration of 100 µM in HepG2 cells cultivated in DMEM (1x) with Galactose in media.
**Diluted in cell media

Outcome: The response in each of the cytoxicity markers described above are normalized to control cells and plotted against the compound concentration. A reference range for each marker is established based on the response in the same marker in control cells (mean ±3×SD) and used to establish the lowest effective concentration (LEC) values for a compound where the cell marker begins to show a response outside the mean ±3×SD of control values. IC_{50}/AC_{50} values are also determined for Nuclei counts and MMP.

Based on the lowest LEC value found with a compound among the above cytotoxicity, a highest safe total Cmax is estimated using a safety factor of 100, calculated as lowest LEC/100. Since the clinical total Cmax is rarely known pre-IDP, K_i or IC_{50} is often used to estimate a TI for cytotoxicity.

Important Information: Compared with the Quadprobe Assay previously run on a Cellomics Array Scanner, the present Quadprobe uses a new platform for assessing cell responses in this assay. The assessment of MMP and Mitochondria Area is now more precise. In addition, the membrane integrity parameter is also more precisely determined due to better dynamic range of the analyzer. Therefore, MMP, mitochondrial mass and Membrane Integrity results produced for a given compound with the present assay can for some compounds be different from previous results produced with the first version of the QuadProbe assay.

hERG Patch Clamp

Purpose: To test if compounds show affinity to the hERG channel

Protocol: Compounds are tested at WuXi according to Work Order 53 using the following protocol:

hERG IonWorks assays are conducted at room temperature in population patch clamp mode. The seal test, cell access, voltage protocols and scan parameters are established with IonWorks Software 2.0.3.4 (Molecular Devices).

Single addition of 3.5 µl of the testing solution is applied and the exposure of testing solution is no less than 5 minutes. The recording for the whole process must pass the quality control or the well will be abandoned, and the compound will be retested (seal resistance must be within the range of 20 ~ 1500 Mega Ohms and hERG current amplitude must be greater than 400 pA).

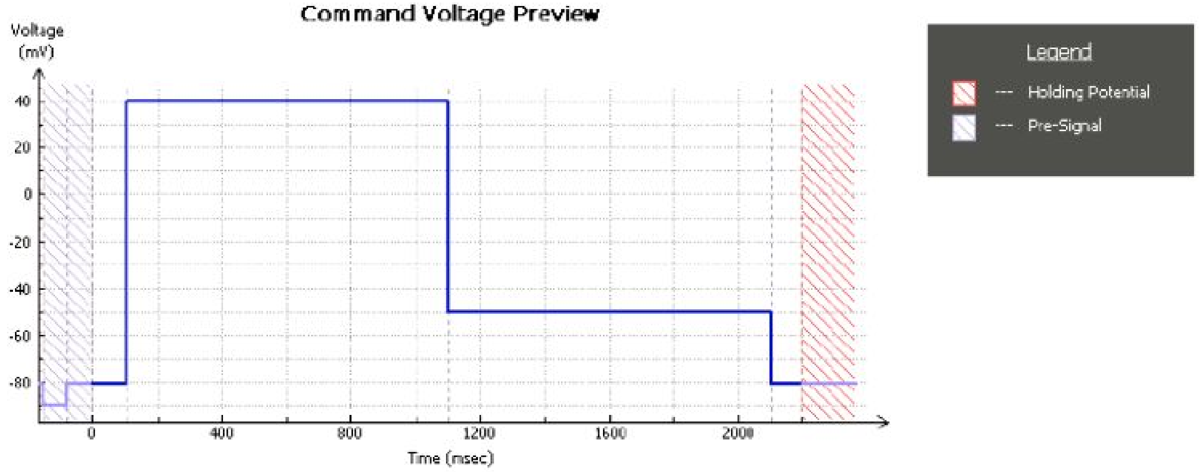

Figure S2. Voltage protocole for hERG IonWorks testing.

Solutions:

External solution (mM): Dulbecco's Phosphate Buffered Saline (DPBS+) with $MgCl_2$ and $CaCl_2$ (sigma, D8662). Kept at 4 ºC.

Internal solution (mM): KCl 140, $MgCl_2$ 2, EGTA 5, HEPES 10, pH to 7.2 with 1*N* KOH, osmolarity to ~275 mOsm. Filtered and kept at 4 °C.

Voltage command protocol: From the holding potential of -80 mV, the voltage is first stepped to +40 mV for 1000 ms to open hERG channels. After that, the voltage is stepped back down to -50 mV for 1000 ms, causing a "rebound" or tail current. The peak amplitude of the tail current is measured and collected for data analysis. Finally, the voltage is stepped back to the holding potential (-80 mV, 100 ms). This voltage protocol is applied before compound application (control), and five minutes after compound application.

Data Analysis: Data analysis is carried out using IonWorks Software 2.0.3.4 (Molecular Devices), Excel 2003 (Microsoft) and GraphPad Prism 5.0 (GraphPad Software, La Jolla, CA).

The percent inhibition values for each test article concentration will be calculated as follows:

{1- (peak current measured after compound perfusion / peak current measured before compound perfusion) / (peak current measured after vehicle perfusion / peak current measured before vehicle perfusion)} x 100%

IC_{50} values will be determined from dose-response curves which are obtained with the sigmoid dose-response (variable slope) equation in GraphPad Prism 5.0, as shown below:

Y=Bottom + (Top-Bottom)/(1+10^((LogIC_{50}-X)*HillSlope))

Where Y represents the percentage of inhibition, X is the logarithm of concentration, IC_{50} is the concentration of compound at 50% inhibition, Top is 100% and Bottom is 0%.

Assay format:

Negative control: 0.1% DMSO

Positive control: Amitriptyline

Test article concentrations: The compound studies will be run at 4 concentrations.

Standard concentrations are given in Annex AX

Experiment model on IonWorks; Population Patch Clamp

Replicates: n=4

Cells: CHO-K1 cells stably expressing hERG channel

Raw data provided by WuXi is copied into a QC and Result workbook created by Exploratory Toxicology. IC50 values are calculated using XLfit.

Bi-directional Permeability Assay in MDR1-MDCK II Cells

MDCKII-MDR1 permeability assay: Bidirectional transport in MDCKII cells transfected with human MDR1 was assessed according to previously published methodology.[4] In brief, cells obtained from the Netherlands Cancer Institute were maintained at 37 °C in α-MEM containing 10% FBS, 100 µg/mL penicillin-G, 100 µg/mL streptomycin, 1% nonessential amino acid under culture conditions of 5% CO_2 and 95% relative humidity.

Transport of test compound (0.5 µM, 0.4% DMSO final concentrations) across the cell monolayer was determined in triplicate on a single test occasion along with controls for low and high permeability (fenoterol and metoprolol, 2 µM) and P-gp efflux (digoxin, 10 µM). Each compound was loaded onto either the apical side (75 µL) or basolateral side (275 µL) with transport buffer (1% BSA in HBSS with 10 mM HEPES (pH 7.4)) on the opposing side of the cells (e.g. 50 µL or 250 µL on the apical or basolateral side). A sample (25 µL) from the donor compartment was taken 30 s after test compound was loaded onto the plate, resulting in a final incubation volume of 50 µL and 250 µL on apical and basolateral sides respectively. At the end of the incubation period, samples (75 µL) were taken from both sides. The donor samples (25 µL) were firstly diluted with transport buffer (50 µL) and then all samples were quenched in acetonitrile (125 µL) containing internal analytical standards.

After centrifugation (20 min, 3220 × g, 4 °C) the supernatants were analyzed by LC–MS/MS. The apparent permeability coefficient (P_{app}) and efflux ratio (ER) were calculated using the equations below:

$$P_{app} = (dC_r/dt) \times V_r /(A \times C_0)$$

$$ER = P_{app}\ A\text{-}B / P_{app}\ B\text{-}A$$

where dC_r/dt is the compound concentration in the receiver chamber as a function of time (µM/s); V_r is the solution volume in the receiver chamber; A is the surface area of the cell monolayer; C_0 is the initial

concentration in the donor compartment; and P_{app} A-B and P_{app} B-A refer to the apparent permeabilities in the respective directions. Compound permeability was classified as low, moderate or high according to P_{app} value binning classifications and the ER was employed to classify compounds as unlikely, possible or likely P-gp substrates (**Table S8**).

Table S8. Criteria and classification for test compounds.

Parameters	Classification	Criteria
Permeability*	Low	P_{app}(AB) ≤ 1.0 (×10^{-6} cm/s)
	Moderate	1.0 < P_{app}(AB) < 5.5 (×10^{-6} cm/s)
	High	P_{app}(AB) ≥ 5.5 (×10^{-6} cm/s)
P-gp Substrate**	Yes	Efflux Ratio ≥ 2

Rat Amphetamine-Induced Hyperlocomotion (AHL) Protocol

Male Harlan Sprague Dawley rats with a mean body weight of 240 g (range 270-302 g) were tested in SmartFrame Open Field locomotor activity test chambers to automatically record locomotor activity. All rats were habituated in locomotor activity enclosures for 30 min, followed by pretreatment by oral gavage for an additional 30 min with either vehicle or a dose of **VU6025733** or the comparator M_4 PAM **VU0467154**. Next, rats were injected subcutaneously with vehicle or a dose of 0.75 mg/kg amphetamine and then monitored for an additional 60 min. Changes in locomotor activity were recorded for a total of 120 min. Locomotor data were expressed as the number of photobeam breaks/5 min intervals across the 120-min test session or as the total ambulation, calculated as sum of photobeam beam breaks from the time of amphetamine administration (60 min) until the end of the study (120 min). Time course data were analyzed by two-way ANOVA with main effects of treatment and time; changes in total ambulation were analyzed by one-way ANOVA followed by Dunnett's *post hoc* test (GraphPad Prism 7 [GraphPad Software, San Diego, CA]). For all tests, $\alpha \leq 0.05$ was considered to represent statistical significance. Finally, percent reversal data were calculated in Microsoft Excel using the following formula: Percent Reversal = 100 - {[(total ambulation in individual animal from t = 60 to t = 120) / (mean total ambulation from t=60 to t=120 in the VAMP group)] * 100}. Mean percent reversal ± S.E.M. was calculated for each dose group using GraphPad Prism 7.

At the end of this behavioral study, each rat was euthanized, then decapitated, and the plasma and brain tissues were collected for the evaluation of exposure levels of **VU6025733** or **VU0467154** by pharmacokinetic analysis.

References

1) Moehle M.S.; Bender A.M.; Dickerson J.W.; Foster D.J.; Qi A.; Cho H.P.; Donsante Y.; Peng W.; Bryant Z.; Stillwell K.J.; Bridges T.M.; Chang S.; Watson K.J.; O'Neill J.C.; Engers J.L.; Peng L.; Rodriguez A.L.; Niswender C.M.; Lindsley C.W.; Hess E.J.; Conn P.J.; Rook J.M. Discovery of the First Selective M_4 Muscarinic Acetylcholine Receptor Antagonist with *in Vivo* Antiparkinsonian and Antidystonic Efficacy. *ACS Pharmacol Transl Sci.* 2021, 4, 1306-1321.
2) Lin J.H.; Chiba M.; Balani S.K.; Chen I.W.; Kwei G.Y.; Vastag K.J.; Nishime J.A. Species differences in the pharmacokinetics and metabolism of indinavir, a potent human immunodeficiency virus protease inhibitor. *Drug Metab. Dispos*. 1996, 24, 1111-1120.
3) Persson M.; Løye A.F.; Mow T.; Hornberg J.J. A high content screening assay to predict human drug-induced liver injury during drug discovery. *J. Pharmacol. Toxicol. Methods*. **2013**, 68, 302-13.
4) Langthaler K.; Jones C.R.; Saaby L.; Bundgaard C.; Brodin B. Application of a new MDCKII-MDR1 cell model to measure the extent of drug distribution in vitro at equilibrium for prediction of in vivo unbound brain-to-plasma drug distribution. *Fluids Barriers CNS*. 2024, 21, 11.
